# The role of brain white matter in depression resilience and response to sleep interventions

**DOI:** 10.1093/braincomms/fcad210

**Published:** 2023-08-02

**Authors:** Tom Bresser, Jeanne Leerssen, Stefanie Hölsken, Inge Groote, Jessica C Foster-Dingley, Martijn P van den Heuvel, Eus J W Van Someren

**Affiliations:** Department of Sleep and Cognition, Netherlands Institute for Neuroscience, 1105 BA, Amsterdam, The Netherlands; Department of Integrative Neurophysiology, Center for Neurogenomics and Cognitive Research (CNCR), Amsterdam Neuroscience, Vrije Universtiteit Amsterdam, 1081 HV, Amsterdam, The Netherlands; Department of Clinical Genetics, Amsterdam Neuroscience, VU University Medical Center, 1081 HV, Amsterdam, The Netherlands; Department of Sleep and Cognition, Netherlands Institute for Neuroscience, 1105 BA, Amsterdam, The Netherlands; Department of Integrative Neurophysiology, Center for Neurogenomics and Cognitive Research (CNCR), Amsterdam Neuroscience, Vrije Universtiteit Amsterdam, 1081 HV, Amsterdam, The Netherlands; Department of Sleep and Cognition, Netherlands Institute for Neuroscience, 1105 BA, Amsterdam, The Netherlands; Institute of Medical Psychology and Behavioral Immunobiology, University Hospital Essen, University of Duisburg Essen, 45122, Essen, Germany; Computational Radiology and Artiﬁcial Intelligence (CRAI), Division of Radiology and Nuclear Medicine, Oslo University Hospital, 0372, Oslo, Norway; Department of Radiology, Vestfold Hospital Trust, 3116, Tønsberg, Norway; Department of Sleep and Cognition, Netherlands Institute for Neuroscience, 1105 BA, Amsterdam, The Netherlands; Department of Clinical Genetics, Amsterdam Neuroscience, VU University Medical Center, 1081 HV, Amsterdam, The Netherlands; Department of Sleep and Cognition, Netherlands Institute for Neuroscience, 1105 BA, Amsterdam, The Netherlands; Department of Integrative Neurophysiology, Center for Neurogenomics and Cognitive Research (CNCR), Amsterdam Neuroscience, Vrije Universtiteit Amsterdam, 1081 HV, Amsterdam, The Netherlands; Department of Psychiatry, Vrije Universtiteit Amsterdam, Amsterdam UMC, 1081 HV, Amsterdam, The Netherlands

**Keywords:** insomnia, depression, cognitive behavioural therapy, circadian rhythm, brain imaging

## Abstract

Insomnia poses a high risk for depression. Brain mechanisms of sleep and mood improvement following cognitive behavioural therapy for insomnia remain elusive. This longitudinal study evaluated whether (i) individual differences in baseline brain white matter microstructure predict improvements and (ii) intervention affects brain white matter microstructure. People meeting the Diagnostic and Statistical Manual of Mental Disorders-5 criteria for Insomnia Disorder (*n* = 117) participated in a randomized controlled trial comparing 6 weeks of no treatment with therapist-guided digital cognitive behavioural therapy for insomnia, circadian rhythm support or their combination (cognitive behavioural therapy for insomnia + circadian rhythm support). Insomnia Severity Index and Inventory of Depressive Symptomatology-Self Report were assessed at baseline and followed up at Weeks 7, 26, 39 and 52. Diffusion-weighted magnetic resonance images were acquired at baseline and Week 7. Skeletonized white matter tracts, fractional anisotropy and mean diffusivity were quantified both tract-wise and voxel-wise using tract-based spatial statistics. Analyses used linear and mixed effect models while correcting for multiple testing using false discovery rate and Bonferroni for correlated endpoint measures. Our results show the following: (i) tract-wise lower fractional anisotropy in the left retrolenticular part of the internal capsule at baseline predicted both worse progression of depressive symptoms in untreated participants and more improvement in treated participants (fractional anisotropy × any intervention, *P*_FDR_ = 0.053, *P*_corr_ = 0.045). (ii) Only the cognitive behavioural therapy for insomnia + circadian rhythm support intervention induced a trend-level mean diffusivity decrease in the right superior corona radiata (*P*_FDR_ = 0.128, *P*_corr_ = 0.108), and individuals with a stronger mean diffusivity decrease showed a stronger alleviation of insomnia (*R* = 0.20, *P* = 0.035).

In summary, individual differences in risk and treatment-supported resilience of depression involve white matter microstructure. Future studies could target the role of the left retrolenticular part of the internal capsule and right superior corona radiata and the brain areas they connect.

## Teaser

Insomnia is a key risk for depression. In a 1-year follow-up study in 117 people with insomnia disorder, Bresser *et al*. show that individual differences in brain white matter play a role in resilience to depression. Moreover, a risk-mitigating sleep intervention can elicit subtle local changes in white matter.

## Graphical Abstract

**Graphical Abstract:**
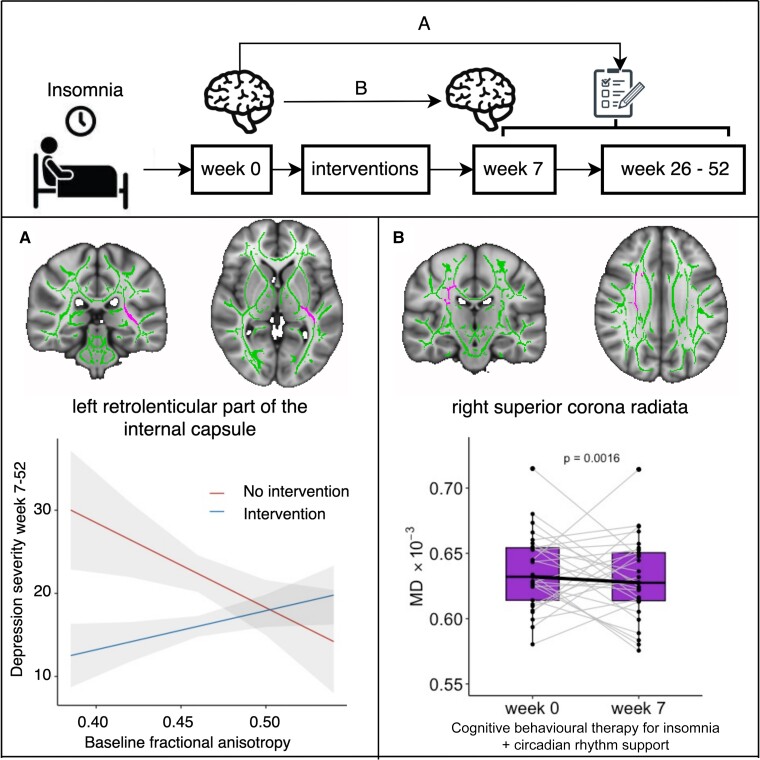

## Introduction

Insomnia disorder is the most common sleep disorder and may be the most common neuropsychiatric disorder as well, surpassed only by the summed prevalence of all anxiety disorders.^1^ About 10% of the general population suffers from insomnia disorder, from here on ‘insomnia’.^1–3^ While good sleepers may experience a bad night once in a while, people suffering from insomnia experience difficulty initiating sleep, difficulty staying asleep and/or early morning awakening at least three nights a week for a period of more than 3 months.^4^ As a result, they experience difficulties with daytime functioning and a reduced quality of life. Moreover, insomnia is a risk factor for other mental disorders including major depressive disorder (MDD).^5^ Several subtypes of people suffering from insomnia have an increased lifetime risk of depression.^6^ The first-line treatment is cognitive behavioural therapy (CBT) specifically for insomnia (CBT-I).^7^ By training patients to change cognitions and behaviours, CBT-I effectively reduces insomnia severity, while it also alleviates depressive symptoms.^8–10^ Clinical effects of CBT-I and other sleep interventions have been studied extensively, but the role of brain white matter (WM) microstructure in the risk and resilience of developing depression, as well as how interventions affect WM microstructure, remains elusive.

Several neuroimaging studies have compared good sleepers and people suffering from insomnia to better understand the underlying mechanisms of insomnia.^11–16^ A review on neuroimaging studies in insomnia and MDD indicates overlap in brain circuits involved,^17^ suggesting that deviations are not necessarily specific to one disorder. In addition to the growing understanding of insomnia-related brain regions and their connections, we can consider structural brain properties, like WM microstructure, as predisposing factors for sleep- and mood-related changes and treatment success. WM comprises the myelinated axons interconnecting (sub)cortical grey matter areas, alongside various non-neuronal cells such as astrocytes and oligodendrocytes.^18^ WM microstructure refers to the organization of this tissue at the microscale and can be quantified using diffusion tensor imaging (DTI)–derived proxy measures, such as fractional anisotropy (FA) or mean diffusivity (MD).^19^ Using longitudinal data, several studies have investigated whether baseline WM microstructure features predicted CBT effectiveness in obsessive–compulsive disorder^20^ and post-traumatic stress disorder.^21^ Similar studies could provide clues on brain circuits involved in insomnia and recovery from insomnia, as well as brain circuits involved in the risk of, and resilience to, worsening of depressive symptoms.

From a treatment perspective, the induction of changes in cognition and behaviour can alter brain WM.^22,23^ Neuroimaging studies have reported changes in WM microstructure following specific training paradigms in motor,^24,25^ tactile^26^ and memory domains.^27,28^ These findings suggest that WM microstructure, quantified using DTI, dynamically responds to specific training and learning protocols, across different timescales. CBT can be conceptualized as training and learning with a clinical purpose. While some studies have found CBT to alter brain function,^29,30^ few studies have investigated CBT effects on brain structure, notably WM microstructure.^20,31,32^ Based on this handful of studies, CBT-induced changes in WM microstructure remain elusive. More longitudinal studies combining treatment with pre- and post-DTI measures including a control group are needed to further our understanding of treatment effects on WM microstructure and how baseline WM microstructure relates to the progression of symptoms and the success of treatments in recovering from symptoms. Pinpointing such effects and predictors could subsequently improve our understanding of the brain circuits involved in resilience and recovery of mental disorders.

The present study aimed to investigate the role of WM microstructure in the progression of insomnia and depressive symptoms, and the effect of interventions on insomnia. We addressed two research questions. First, can individual differences in baseline WM microstructure predict the progression of insomnia and depressive symptoms, and the effect of treatments on this progression? Second, can 6 weeks of insomnia interventions elicit WM microstructure changes in people suffering from insomnia? Of note, since the two questions do not necessarily have to involve the same WM tracts, they require separate analyses. We expected our repeated measures design with multiple interventions including a no treatment (NT) group to reveal treatment response–related WM microstructure features that could provide clues on brain circuits involved in insomnia and recovery from insomnia, as well as brain circuits involved in the risk of, and resilience to, worsening of depressive symptoms.

## Materials and methods

### Procedure

This WM microstructure study was part of planned investigations (https://erc.easme-web.eu/? p = 671084#) integrated in a randomized controlled trial of which the clinical findings have been reported.^10^ In brief, we measured WM microstructure using DTI both at baseline and after 6 weeks of either NT or therapist-guided digital CBT-I, circadian rhythm support (CRS) or combined CBT-I + CRS. The Insomnia Severity Index^33^ (ISI) and Inventory of Depressive Symptomatology-Self Report^34^ (IDS-SR) were assessed directly before (T0) and after (T1) the interventions and followed up at 6, 9 and 12 months (T2–4). To restrain the number of tests performed in our analyses, we focus on FA and MD to reliably asses WM microstructure in the different tracts.^19,35^

### Participants

Participants were people diagnosed with insomnia according to the Diagnostic and Statistical Manual of Mental Disorders, 5th edition^4^ criteria. Inclusion criteria were as follows: (i) insomnia diagnosis according to the Diagnostic and Statistical Manual of Mental Disorders, 5th edition, and the International Classification of Sleep Disorders, 3rd edition^36^; (ii) an ISI score^33^ ≥10; and (iii) an age between 18 and 70 years. Furthermore, focusing on mitigating the risk of developing depression, we selected only people that suffered from insomnia of a subtype that conveys a high risk of developing depression.^6^ In brief, participants filled out the Insomnia Type Questionnaire to identify their profile of relatively stable traits, which can be used to categorize people suffering from insomnia with one of five subtypes. We included the highly distressed subtype, moderately distressed reward sensitive subtype and moderately distressed reward insensitive subtype (see Blanken *et al.*^6^) that are characterized by one or more of the following traits: negative affect, rumination, reduced subjective happiness, pre-seep arousal, insomnia response to stress and reduced positive affect. Exclusion criteria were as follows: (i) current diagnosis of MDD; (ii) current treatment with antidepressant medication; (iii) current CBT-I treatment; (iv) a severe sleep disorder other than insomnia (i.e. sleep apnoea, restless leg syndrome and periodic limb movement disorder); (v) a current diagnosis of any other severe psychiatric or neurological disorder; (vi) severe physical or mental impairment due to stroke or traumatic head injury; (vii) night work or rotating shift work; (viii) a known eye condition incompatible with light exposure; (ix) a history of light-induced migraine or epilepsy or severe side effects to bright light; or (x) MRI contraindications. Participants were screened prior to enrolment using online questionnaires, which included questions to exclude people diagnosed with a severe sleep disorder other than insomnia (i.e. sleep apnoea, restless leg syndrome and periodic limb movement disorder). In addition, a polysomnogram recorded at baseline was inspected on undiagnosed severe sleep disorders other than insomnia. Participants were recruited through media advertisements, the Netherlands Sleep Registry (www.slaapregister.nl) and flyers. The study was performed in accordance with the declaration of Helsinki. The protocol^37^ was approved by the Medical Ethics Committee of the VU University Medical Centre (NL63139.029.17) and pre-registered on the International Clinical Trial Registry Platform (https://trialsearch.who.int/Trial2.aspx?TrialID=NTR7567). Investigation of brain structural differences and changes was part of the planned analyses integrated with the clinical trial (https://erc.easme-web.eu/?p=671084#). Participants provided written informed consent.

### MRI acquisition and processing

MRI acquisition was part of a randomized control trial of which the clinical findings have been reported.^10^ From the original sample, 15 participants were excluded due to MRI contraindications, excessive head motion or otherwise incomplete MRI data (Supplementary Fig. 1). DTI and T_1_-weighted imaging assessments were performed in the week before onset of the interventions (Week 1, T0, baseline) and repeated 6 weeks later, immediately after completion of the interventions (Week 7, T1). Participants were scanned at the Spinoza Centre for Neuroimaging (Amsterdam, The Netherlands) and instructed to refrain from caffeinated beverages, alcohol and drugs on assessment days. T_1_-weighted scans were pre-processed and segmented using the Freesurfer^38^ stable version 6.0.1 ‘recon-all’ function. The resulting segmentation and original T_1_-weighted scan were used to pre-process the DTI data using the Connectivity Analysis Toolbox^39^ (CATO, version 3.1.2) generating the FA and MD images. In order to address within-subject residual variation across timepoints in repeated measures, we implemented a half-way linear transformation between the T0 and T1 images to optimize longitudinal alignment.^40^ The resulting FA base template for each subject was used in the subsequent tract-based spatial statistics^41^ to generate a study-specific FA skeleton representing all major WM tracts (see Supplementary Methods for more details).

### Sleep interventions

Participants were randomized to one of four interventions: NT, therapist-guided digital CRS, therapist-guided digital CBT-I or combined CBT-I + CRS. All three interventions were provided online guided by trained therapists and addressed five topics. The intervention is briefly described in the Supplementary Methods and has been documented in full before.^10,37^

### Assessment of insomnia and depression severity

The ISI^33^ and IDS-SR^34^ were used to assess symptom severity prior to the intervention (T0, baseline) and in Week 7 immediately after the intervention (T1, post-treatment), aligned with the DTI assessments. The clinical variables were moreover assessed at three additional follow-ups in Week 26 (T2), Week 39 (T3) and Week 52 (T4). ISI scores range from 0 to 28, with higher scores indicating more severe insomnia. IDS-SR scores range from 0 to 84, with higher scores indicating more severe depressive symptoms. The effects of NT, CBT-I, CRS and CBT-I + CRS on ISI and IDS-SR across timepoints, as estimated with mixed effect regression analysis, have been described in detail elsewhere^10^ and were nearly identical in the current subsample of participants who completed the two MRI sessions with success.

### Statistical analysis

All skeletonized WM tracts described in the International Consortium of Brain Mapping-DTI-81 WM label atlas^42,43^ by John Hopkins University were quantified, except for the left and right tapetum. Mean FA and MD were calculated by averaging all voxels belonging to the skeletonized tract. In addition, we included the whole skeleton mean and mean value of all peripheral voxels, defined as voxels without International Consortium of Brain Mapping-DTI-81 label, resulting in 48 ‘tracts’. Within each separate tract, extreme outliers defined as values three times the interquartile range above the third quartile or below the first quartile were determined using the ‘rstatix’ package^44^ version 0.7.0 and excluded. As a result, sample sizes differ slightly per tract and are reported in the ‘Results’ section. Tract-wise outcomes were analysed using linear and linear mixed effect regression models using the LME4 package^45^ version 1.1-30 in R^46^ version 4.0.4. All *P*-values were corrected for multiple testing using false discovery rate (FDR) correction.^47^ In addition to FDR correction, we calculated a Bonferroni-based correction for multiple, correlated endpoints^48^ (*P*_corr_) given the between-tract correlation of FA values and of MD values. This method uses the interclass correlation to adjust for correlated measures, here tracts. The single fixed rater interclass correlation coefficient (ICC) of MD (ICC_MD_ = 0.162) and FA (ICC_FA_ = 0.292) at Week 0 (T0) was determined using the ‘psych’ package^49^ version 2.2.5.

To assess the effect of baseline WM microstructure on improvements in insomnia and depressive symptom severity after treatment, we utilized all follow-up measures of ISI and IDS-SR in a mixed effect analysis with age, sex and the baseline measure as covariate (e.g. ISI_T1–4_ ∼ group + tract_FA_T0_ + group:tract_FA_T0_ + ISI_T0_ + age + sex) and a random intercept for each subject. Subsequently, we combined CRS, CBT-I and CRS + CBT-I into a single ‘intervention’ group and repeated the analysis to capture general intervention by tract interaction effects on ISI and IDS-SR. Linear mixed effect model *P*-values were estimated using the Satterthwaite degrees of freedom method by the lmerTest package^50^ version 3.1-3.

To assess the effect of insomnia interventions on WM microstructure, we used a linear regression model for each tract and tested whether the three active intervention groups differed significantly from the NT group at post-treatment (T1) while correcting for age, sex and corresponding baseline values (e.g. FA_T1_ ∼ CBT-I + CRS + ‘CBT-I + CRS’ + age + sex + FA_T0_). Reported standardized regression coefficients (*β*) were obtained by scaling all numeric variables before fitting the regression model. We controlled for the potential confounding effect of current medication use by rerunning all analyses that yielded significant effect, now adding categorical covariates for antidepressants, antianxiety drugs, antihypertensives, thyroid medication, antiasthmatics, headache medicines, stimulants, sleep medicines and other medication (see Supplementary Table 2 for an overview).

Tract-wise analyses were complemented by voxel-wise analysis to investigate whether improvements in insomnia and depressive symptom severity after treatment were associated with baseline WM microstructure in specific parts of the tracts or more likely distributed across a tract or heterogeneous between subjects, i.e. not requiring sample convergence at the voxel level.^51^ Voxel-wise analyses included age and sex as covariates (see Supplementary Methods for more details).

## Results

### Demographics

DTI was successfully assessed at both T0 and T1 in 117 participants. Participants included in the current analyses had a mean age of 48.1 (SD = 13.1, *range* 21–69). At T0, the ISI mean score was 15.8 (SD = 3.7, *range* 5–25), the mean IDS-SR score was 19.0 (SD = 7.1, *range* 4–38) and the mean duration of their insomnia was 9.3 years (SD = 10.5, *range* 0.1–45.8, *n* = 113). At baseline, participants randomized to the four interventions did not significantly differ regarding age, sex, years of education, alcohol consumption, smoking, handedness, ISI, insomnia duration, depression symptoms (IDS-SR), anxiety (Beck Anxiety Inventory), occurrence of comorbidities and medication(s) use (Table 1; Supplementary Tables 1 and 2). As to be expected when comparing randomized groups on a large number of variables, some group differences reached significance. As shown in Supplementary Table 1, unemployment was more prevalent in the NT group, and the Positive and Negative Affect Schedule negative affect score was lower in the CRS group. The four intervention groups were also assessed on whole brain properties and did not differ significantly in total brain volume, total grey matter volume, cerebral WM volume, skeleton mean FA or skeleton mean MD (*P* > 0.402, Supplementary Table 1).

**Table 1:** Descriptive measures

	CBT-I	CRS	CBT-I + CRS	NT	*P*
*N*	27	31	29	30	
Age in years^a^	48.0 (13.9)	48.9 (13.5)	47.2 (12.6)	48.1 (12.9)	0.973
Sex					0.963
Female^b^	20 (74.1)	23 (74.2)	22 (75.9)	21 (70.0)	
Male^b^	7 (25.9)	8 (25.8)	7 (24.1)	9 (30.0)	
Education in years^a^	16.9 (2.7)	16.7 (2.7)	17.2 (3.2)	15.7 (3.5)	0.282
Comorbidities^b^					0.555
Anxiety disorder	1 (3.7)	0 (0.0)	0 (0.0)	0 (0.0)	
ADHD	0 (0.0)	1 (3.2)	1 (3.4)	0 (0.0)	
Autism	0 (0.0)	0 (0.0)	0 (0.0)	1 (3.3)	
Post-traumatic stress disorder	2 (7.4)	0 (0.0)	0 (0.0)	1 (3.3)	
No comorbidities	23 (85.2)	27 (87.1)	27 (93.1)	26 (86.7)	
Other	1 (3.7)	3 (9.7)	1 (3.4)	2 (6.7)	
Sleep medication users^b^	5 (18.5)	6 (19.4)	5 (17.2)	6 (20.0)	0.994
Mild sleep apnoea^b^	1 (3.7)	1 (3.2)	1 (3.4)	2 (6.7)	0.903
Mild restless leg syndrome^b^	5 (18.5)	3 (9.7)	3 (10.3)	3 (10.0)	0.697
Insomnia duration in years^a,c^	6.0 (7.1)	9.7 (11.6)	6.7 (4.6)	10.9 (11.2)	0.161
Insomnia severity (ISI)^a^	16.0 (4.1)	15.4 (3.0)	16.0 (3.6)	15.8 (4.1)	0.894
Sleep quality (PSQI)^a^	10.8 (3.4)	10.55 (3.2)	10.5 (3.3)	10.1 (3.3)	0.876
Depression severity (IDS-SR)^a^	18.9 (8.1)	18.7 (6.8)	19.3 (7.7)	19.2 (6.2)	0.985
Anxiety (BAI)^a^	7.5 (5.7)	7.7 (4.8)	7.9 (5.7)	7.5 (4.8)	0.989

ADHD, attention-deficit hyperactivity disorder; ISI, Insomnia Severity Index; PSQI, Pittsburgh Sleep Quality Index; IDS-SR, Inventory of Depressive Symptomatology-Self Report; BAI, Beck Anxiety Inventory.

a Mean (SD).

b *n* (%).

c *n*_total_ = 113.

### Insomnia interventions alleviate insomnia and depression severity

A detailed description on the clinical effects of the interventions as assessed in all 132 participants in the randomized controlled trial has been reported elsewhere.^10^ Effects were nearly identical in the current subsample of *n* = 117 (88.6%) with complete DTI. In short, during the follow-up period of 1 year, CBT-I and CBT-I + CRS evoked the strongest reduction in ISI and IDS-SR scores compared to NT. Between-group Cohen’s *d* effect sizes were as follows: CBT-I versus NT, *d*_ISI_ = −1.05, *P* < 0.001, *d*_IDS-SR_ = −0.78, *P* = 0.001, and CBT-I + CRS versus NT, *d*_ISI_ = −0.87, *P* = 0.002, *d*_IDS-SR_ = −1.01, *P* < 0.001. CRS by itself, i.e. given without CBT-I, did not significantly reduce ISI or IDS-SR scores compared to NT (*d*_ISI_ = −0.36, *P* = 0.197; *d*_IDS-SR_ = −0.36, *P* = 0.124).

### The effect of baseline WM microstructure on improvements in insomnia and depressive symptom severity after treatment

**Figure 1:**
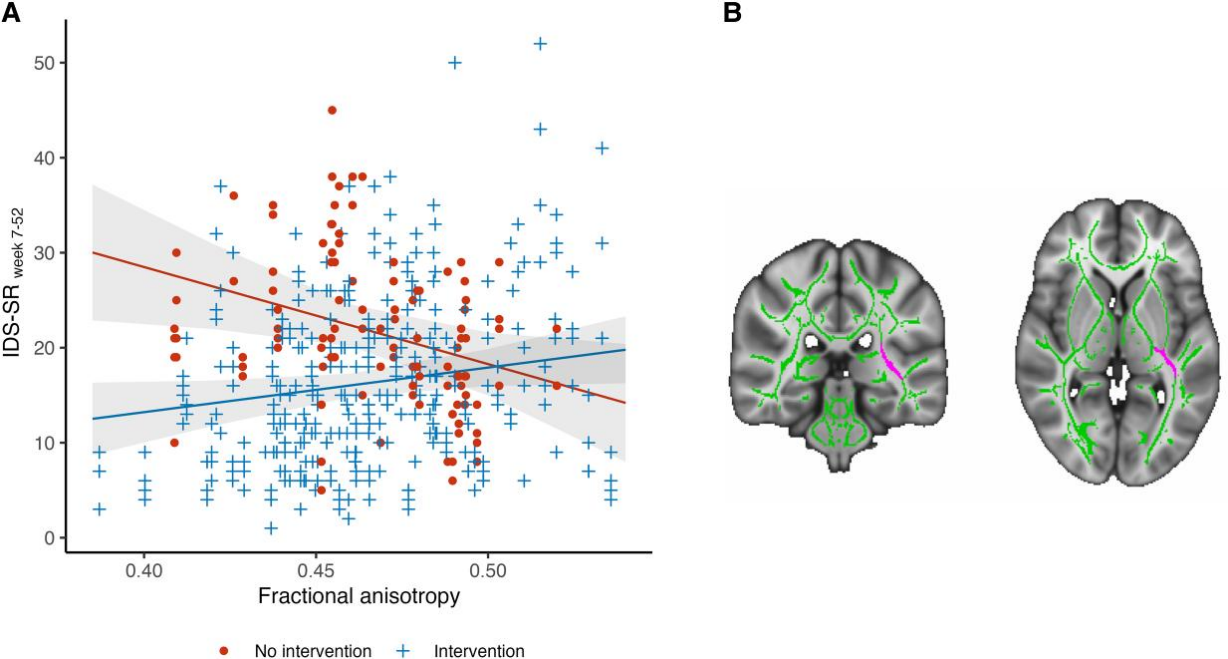
**Baseline FA in the left RLIC predicts depressive symptom severity across follow-ups in the following year**. (**A**) Regression lines visualizing the associations of baseline FA in the left RLIC with IDS-SR across follow-ups as revealed by the linear mixed model [*t*(115.38) = 3.38, *P* = 0.001, *P*_FDR_ = 0.053, *P*_corr_ = 0.045]. The line with the negative slope shows how lower baseline FA in the left RLIC predicts higher IDS-SR across follow-ups in untreated participants. The line with the positive shows how lower baseline FA in the left RLIC predicts a stronger improvement in treated participants. (**B**) MNI152 T1 brain (1 mm, shown at −23, −31, 2) with the left RLIC (magenta) within the study-specific WM skeleton (green). FA, fractional anisotropy; RLIC, retrolenticular part of internal capsule; IDS-SR, Inventory of Depressive Symptomatology-Self Report; MNI, Montreal Neurological Institute.

To assess the predictive value of WM microstructure features, we analysed coefficients of ‘WM microstructure by intervention group’ with IDS-SR and with ISI interactions at follow-up, Weeks 7–52, using mixed effect models while controlling for age, sex and IDS-SR or ISI at baseline. This tract-wise approach revealed 26 FA by intervention group interactions and 8 MD by intervention group interactions with IDS-SR and ISI. While the individual group interactions were no longer significant after correcting for multiple testing (Supplementary Tables 3 and 4), we observed that significant FA by intervention group interactions with IDS-SR at follow-up in the right anterior corona radiata and left retrolenticular part of internal capsule (RLIC) were consistently present across each of the three individual treatments CRS, CBT-I and CRS + CBT-I. Indeed, analyses combining CRS, CBT-I and CRS + CBT-I as one ‘any intervention’ group revealed that baseline FA of the left RLIC (but not right anterior corona radiata, see Supplementary Table 5) was associated with IDS-SR at follow-up in an intervention-dependent manner (FA × any intervention, *B* = 150.2, SE = 44.5, *t*(115.38) = 3.38, *P* = 0.001, *P*_FDR_ = 0.053, *P*_corr_ = 0.045). We did not observe a similar effect for ISI (FA × any intervention, *B* = −12.2, SE = 24.7, *t*(115.38) = −0.49, *P* = 0.62, Supplementary Table 5). Investigation of slopes showed that in the NT group (*n* = 30), lower FA was associated with higher IDS-SR at follow-up (Fig. 1, *B* = −102.5, SE = 40.6). In contrast, among participants receiving any intervention (*n* = 87), lower FA was associated with lower IDS-SR at follow-up (*B* = 47.7, SE = 20.5). The intervention-dependent effect remained significant after correcting for current medication use (FA × any intervention, *B* = 163.7, SE = 41.8, *t*(116.6) = 3.92, *P* = 0.0002).

### The effect of insomnia interventions on WM microstructure

**Figure 2:**
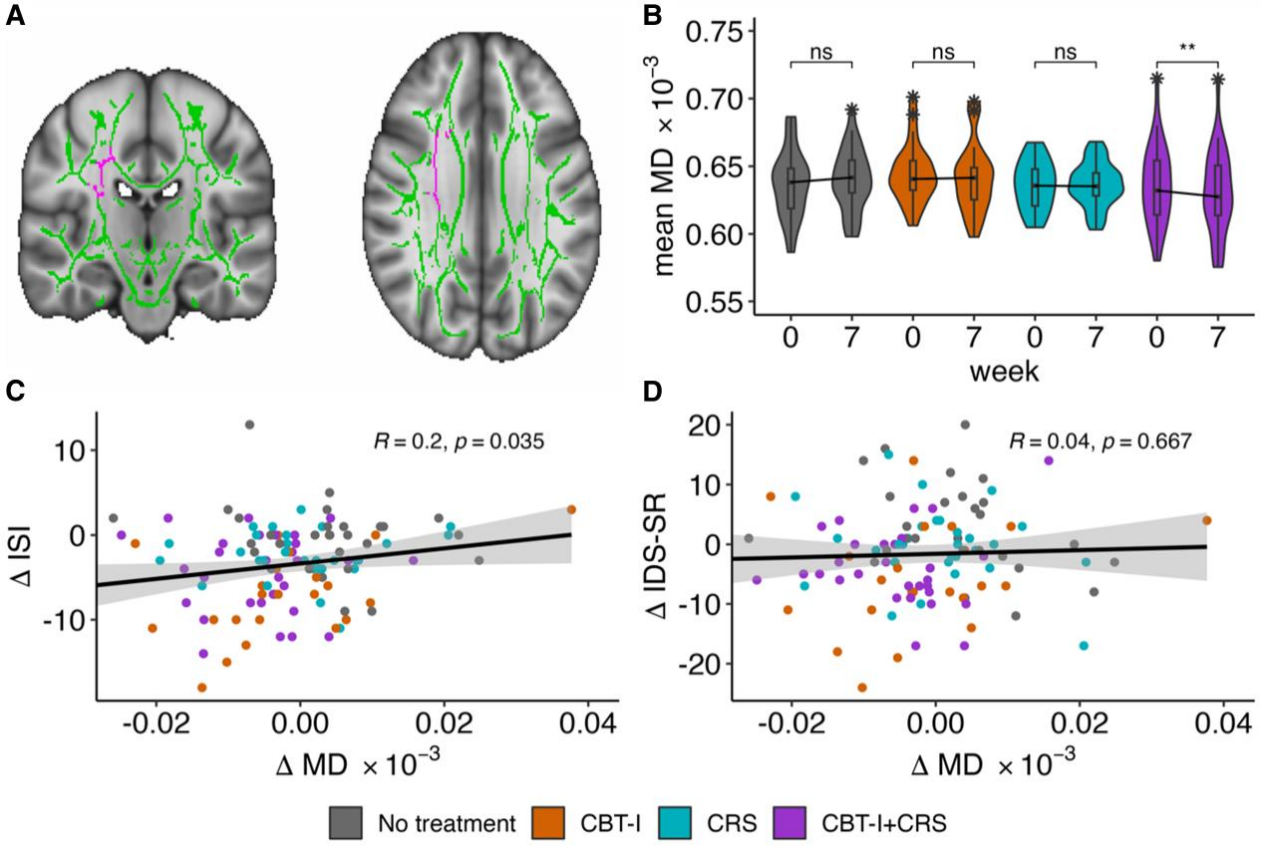
**MD in the right SCR changes following CBT-I combined with CRS**. (**A**) T1 MNI152 brain (1 mm, shown at −3, −19, 30) showing the right SCR within the study-specific WM skeleton. (**B**) Violin plots of right SCR MD for each group at baseline (T0) and Week 7 (T1). Paired *t*-test significance within groups is above the distributions, indicating that right SCR MD decreases only in the CBT-I + CRS group. **t(28) = 3.5, *P* ≤ 0.01. (**C**) Correlation between T0-to-T1 change in right SCR MD and T0-to-T1 ISI score. (**D**) Correlation between T0-to-T1 change in right SCR MD and T0-to-T1 IDS-SR score. MD, mean diffusivity; SCR, superior corona radiata; CBT-I, cognitive behavioural therapy for insomnia; CRS, circadian rhythm support; IDS-SR, Inventory of Depressive Symptomatology-Self Report; ISI, Insomnia Severity Index; MNI, Montreal Neurological Institute.

To investigate treatment effects on WM microstructure, we compared the intervention groups CBT-I, CRS and CBT-I + CRS with the NT group while correcting for age, sex and corresponding baseline measure. Tract-wise regression analyses on FA and MD means across 48 WM tracts found several effects (see Supplementary Tables 6 and 7). The combined treatment, CBT-I + CRS, was of interest as it elicited a significant reduction in MD from T0 to T1 in the right superior corona radiata (SCR, Cohen’s *d* = −0.65, *t*(28) = −3.49, *P* = 0.003, *n*_CBT-I + CRS_ = 29). The decrease was significantly stronger compared to the change in the NT group (Fig. 2, *β* = −0.336, SE = 0.109, *t*(105) = −3.08, *P* = 0.003, *n*_total_ = 112). While this effect became non-significant after FDR correction (*P*_FDR_ = 0.128) and Bonferroni for correlated endpoint measures (*P*_corr_ = 0.108), it remains of interest because independent of intervention group, individual differences in the T0-to-T1 decrease in MD in the right SCR were significantly correlated with individual differences in the corresponding decrease in insomnia severity assessed with ISI_T1–T0_ (*r* = 0.20, *P* = 0.035). The association was only present for insomnia severity and not for depressive symptoms (IDS-SR_T1–T0_, *r* = 0.04, *P* = 0.667, Fig. 2). Ancillary analysis showed that the reduction in MD remained significant after correcting for current medication use (SCR, Cohen’s *d* = −0.28, *t*(28) = −2.50, *P* = 0.014, *n*_CBT-I + CRS_ = 29). Voxel-wise analysis permutation tested with threshold-free cluster enhancement did not yield any specific cluster(s) that significantly differed between the intervention groups and NT.

## Discussion

In this study, we addressed the role of brain WM in the effect of cognitive, behavioural and environmental treatments for insomnia on the severity of insomnia and depressive symptoms. In order to address the questions in the most sensitive way, we recruited people suffering from insomnia of a subtype that conveys a high risk of developing depression. We applied complementary methodological approaches, i.e. tract-wise and voxel-wise analyses, to evaluate (i) the role of individual differences in baseline WM microstructure in treatment-induced improvements in insomnia and depression severity and (ii) the effect of insomnia interventions on brain WM microstructure. As reported before,^10^ CBT-I and CBT-I combined with CRS significantly reduced both depression and insomnia severity relative to changes in the NT group during four follow-ups spanning 1 year.

First, we studied the role of individual differences in baseline WM microstructure in the progression of insomnia and depression severity and treatment-induced changes in their progression. Tract-wise analysis revealed that reduction in IDS-SR at follow-up, Weeks 7–52, was predicted by a treatment group × WM microstructure interaction in the left RLIC, especially when all three interventions were combined in an ‘any intervention’ group. Among participants with insomnia that did not receive any intervention, depressive symptoms worsened in proportion to lower FA in the left RLIC. The finding suggests that higher FA in the left RLIC may protect from worsening of depressive symptoms in people with untreated insomnia. Previous studies have reported similar associations for WM microstructure measures in the RLIC. In older adults, mean FA in the RLIC was associated with depressive symptom severity.^52^ DTI studies have also linked measures of WM microstructure in the RLIC to sleep traits. In MDD, patients with low sleep efficiency (SE, <90%) had clusters within the RLIC with higher axial diffusivity compared to the normal SE group.^53^ Another study in bipolar disorder patients found various associations between sleep traits and FA, MD or RD. Higher RD in the RLIC was associated with shorter time asleep assessed with actigraphy.^54^ Furthermore, DTI measures of WM microstructure within the RLIC, next to other tracts, were genetically correlated to ‘eveningness’.^55^ Such a late chronotype and/or difficulty initiating sleep are indeed associated with the risk of developing depressive disorder.^56^ The precise role and underlying tissue microstructural properties remain unclear and should be addressed in future studies. Taken together, WM microstructure in the RLIC has repeatedly been associated to sleep- and mood-related traits across healthy and disordered populations. Our findings now add that WM microstructure in the left RLIC potentially has a protective effect on depression severity in untreated insomnia.

Our second aim was to study whether WM microstructure was affected by 6 weeks of insomnia interventions, relative to NT. Tract-wise analysis revealed that CBT-I combined with CRS, the treatment that was most demanding and effective, reduced MD in the right SCR, which correlated with improvements in insomnia severity. Compared with other studies reporting changes in WM microstructure after learning and training,^22–25,27,28,40,57–59^ our result is subtle and more diffuse. Protocols used in previous studies elicited strong and targeted effects in specific and spatially restricted brain regions, often the primary cortex. Clinical interventions are very different in this aspect. Given the variety and complexity of the cognitive domains involved in the insomnia interventions, and for that matter all CBT interventions, the activated brain areas are probably more diffuse and WM microstructure responses more subtle at best.^20^

The within-subject decrease of MD in the right SCR after CBT-I combined with CRS compared with NT suggests decreased extracellular space, increased myelination or other changes that reduce water diffusion. Such changes may be interpreted as supporting improved WM microstructure.^60^ The SCR region accommodates corticospinal fibres and projection fibres that connect the frontal and middle cerebral cortex with subcortical grey matter. Frontal regions, such as the anterior cingulate cortex, have been associated with ID.^11,61–63^ Moreover, previous studies suggested differences in WM microstructure in the right SCR of insomnia patients^14^ and MDD patients.^64^ Measures of WM microstructure in the SCR have also been associated with lower SE in MDD^53^ and with shorter total sleep time in healthy adults.^65^ As compared to MDD patients with a relatively normal SE (≥90%), MDD patients with low SE (<90%) showed higher axial diffusivity in several WM clusters, which included the left SCR.^53^ Furthermore, compared to people with a relatively normal total sleep time, short sleepers had lower FA and higher MD in a cluster within the right SCR.^65^ All things considered, the right SCR region fits within an emerging insomnia-related circuit consisting of frontal regions, subcortical regions and underlying WM tracts. The subtle WM microstructural change in the right SCR after CBT-I combined with CRS suggests that insomnia-related deviations in WM microstructure are not static and can change with successful intervention.

Voxel-wise analysis could not specify the tract-wise findings to particular locations within these tracts that were most affected and identically so across participants. The two types of analysis evaluated different possible WM microstructure changes over time. Voxel-wise analysis investigates whether groups differ in changes in a specific cluster within a tract, assuming that it is this particular subpart that is affected most and identically located across participants. Tract-wise analysis on the other hand evaluates whether groups differ in changes in the mean WM microstructure of predefined tracts, allowing for more between-subject heterogeneity of the extent and locations in the tract where changes actually occur. The difference in outcomes of the two types of analysis suggests that CBT-I combined with CRS can affect measures of WM microstructure in the right SCR in a heterogeneous way, rather than in a spatially specific way that is more or less identical for most participants.

Taken together, our results highlight the potential of combined insomnia interventions to elicit subtle changes in local WM microstructure and in parallel how local WM microstructure can contribute to resilience to worsening of depressive symptoms in untreated insomnia. Future studies could explore if microstructural changes in WM can leverage clinical improvements and potentially help develop more effective interventions.

### Strengths and limitations

A particular strength of the study is the longitudinal design combining several interventions and a ‘NT’ control group. Furthermore, we implemented a longitudinal tract-based spatial statistics approach to improve alignment by applying a linear registration between the two timepoints of each subject and applying only one non-linear registration per subject to standard space.^40^ This approach minimizes bias from non-linear registration of separate timepoints to standard space. A number of limitations should be noted. First, most of the effect sizes are small and although the current group sizes are conventional for neuroimaging studies, including those that demonstrated effects of training and learning on WM microstructure, they are likely underpowered to detect small effects with certainty. Replication in larger samples is required to validate and better comprehend these effects. Second, the current study used FA and MD as proxy for WM microstructure. These measures have proven sensitive to detect microstructural changes in WM to some extent.^19^ However, DTI proxy measures alone are insufficient to draw conclusions about biological changes that drive the measured changes or differences in WM diffusion.^60,66^ More sophisticated diffusion analysis methods are becoming available.^18,60^ Improved reproducibility, better inference and increased sensitivity and specificity, especially in regions with crossing fibres, may be expected from these more sophisticated methods and by using multiple modalities.^60,67^

## Conclusion

We have shown that a higher FA in the left RLIC is associated with a better resilience to worsening of depressive symptom severity over time in untreated insomnia. We further show that 6 weeks of CBT for insomnia combined with CRS can elicit a subtle change in WM microstructure within the right SCR. These changes were paralleled by amelioration of insomnia symptoms and potentially reflect altered frontal–subcortical connectivity. In summary, our findings suggest involvement of WM microstructure in the risk and resilience to worsening of depressive symptoms in people with insomnia and suggest that insomnia interventions can elicit subtle, likely heterogeneous or distributed, changes in WM microstructure. Future studies could target the role of the left RLIC and right SCR and the brain areas they connect.

## Supplementary Material

fcad210_Supplementary_Data

## Data Availability

The data that support the findings of this study are available from the corresponding author, upon reasonable request.
