# Correction to: The role of brain white matter in depression resilience and response to sleep interventions

**DOI:** 10.1093/braincomms/fcaf064

**Published:** 2025-02-28

This is a correction to: Tom Bresser *et al.*, The role of brain white matter in depression resilience and response to sleep interventions, *Brain Communications*, Volume 5, Issue 4, 2023, https://doi.org/10.1093/braincomms/fcad210

In the version originally published, the reported *n* of the different therapy groups in Supplementary Figure 1 were misplaced, resulting in a mismatch with Table 1 and Supplementary Table 1 (both of which show the correct *n* values). Supplementary Figure 1 has been updated online. The correction has no further implications on the results or conclusions presented in the article.

