# Supplementary material for: The role of brain white matter in depression resilience and response to sleep interventions
Source: Brain Commun. 2023 Aug 2;5(4):fcad210. doi: 10.1093/braincomms/fcad210 (PMC10406158; doi:10.1093/braincomms/fcad210)
Supplement: fcad210_Supplementary_Data [file fcad210_supplementary_data.pdf]

Supplementary material

Methods

Sleep interventions

Participants were randomized to one of four interventions: no treatment (NT), therapist-guided digital Circadian Rhythm Support (CRS), therapist-guided digital cognitive behavioral therapy for insomnia (CBT-I) or combined CBT-I+CRS. All three interventions were provided online guided by trained therapists. A detailed description of the interventions has been provided elsewhere.[1,2] In brief, the CRS intervention addressed five topics. 1) Psychoeducation about circadian rhythms and light exposure, including the daily use of a Philips EnergyUp HF3430/01 light. Participants were instructed to use the light for 30 minutes shortly after awakening at a distance of 40 cm to the eyes; 2) Consolidating the light routine and schedule at least 30 minutes of physical activity to be performed at a fixed time of day; 3) Consolidating scheduled physical activity and increasing its intensity; 4) Warming the body 2-3 hours before bedtime using a warm bath (temperature of 37- 39°C) or a hot shower, if no bath was available, for approximately 3 times a week; 5) Revisiting the previous schedules to optimize feasibility and secure continued adherence. The CBT-I intervention also addressed five topics 1) Psychoeducation; 2) Stimulus control and sleep restriction; 3) Worrying and relaxation; 4) Erroneous cognitions about sleep; 5) Plans to secure continuation after the intervention period. The combined CBT-I+CRS intervention simultaneously addressed the topics of both CRS and CBT-I described above.

Magnetic resonance imaging (MRI) acquisition

Head motion during the scanning sequence was restricted by foam pads. All scans were acquired on a 3-Tesla MRI scanner with a 32-channel head coil (Philips Achieva, Best, the Netherlands). T1-weighted (T1w) MRI scans were acquired according to the ADNI protocol[3]: repetition time = 6.5ms, echo time (shortest) = 2.9ms, voxel size = $1mm^3$, 211 slices, field of view = 256x256x211mm, flip angle = 9°. Diffusion tensor imaging (DTI) scans were acquired using a multiband spin-echo echo-planar imaging (EPI) sequence using the following parameters: anterior-posterior phase encoding direction, multi-band factor of 2, repetition time = 4683ms, echo time (shortest) = 95ms, voxel size = $(2mm)^3$, 66 transverse sections (slices) without gap, field-of-view = 224x224x132mm, 90° flip angle, 9 non-diffusion weighted (b=0) images and 88 diffusion weighted images (29 with b=1000 s/mm^2, 59 with b=2000 s/mm^2). In addition, we acquired one non-diffusion weighted scan in the opposite phase encoding direction (posterior-anterior) to estimate susceptibility induced distortions (see "preprocessing"). All scans were acquired in the afternoon between 13:00 and 17:00 hr, between December 2018 and November 2019.

Preprocessing

T1w scans were preprocessed and segmented using the Freesurfer[4] stable version 6.0.1 'recon-all' function. The resulting segmentation and original T1w scan were used to preprocess the DTI data using the Connectivity Analysis Toolbox[5] (CATO, version 3.1.2). Part of preprocessing in this package is registering the Freesurfer segmentation to the diffusion reference image enabling T1w-based brain masking and white matter (WM) segmentation. CATO configuration included the correction of the DTI data for eddy currents, subject movement and susceptibility induced distortions using `eddy`[6,7] and `topup`[8,9] from the FMRIB Software Library[8] (FSL version 6.0.4).

Finally, diffusion was reconstructed by CATO using the Diffusion Tensor Imaging (DTI) model and subsequently resulted in a fractional anisotropy (FA) and mean diffusivity (MD) image per subject. FA and MD images were quality controlled visually for artifacts and abnormalities. DTI data of 15 participants were missing or excluded from analyses due to excessive head motion, artifacts or otherwise incomplete MRI data at T0 or T1.

Longitudinal tract-based spatial statistics

Tract-based spatial statistics[10] (TBSS) is a common WM microstructure analysis method to overcome cross-subject alignment issues while performing voxel-wise analysis of FA and MD images. In order to address within-subject residual variation across timepoints in repeated measures studies like ours, some have implemented a half-way linear transformation between timepoints to optimize longitudinal alignment[11]. We adopted a similar approach by performing a linear registration using FLIRT[12] between the T0 and T1 FA image of every participant. Both images were then resampled into a halfway space,[13] minimizing registration bias towards one of the two timepoints. The two halfway FA images were then averaged to create a FA base template for that subject to be used in the subsequent TBSS. Next, all FA base templates were used to generate a study-specific FA skeleton representing all major WM tracts.

FA images were aligned to a 1x1x1 mm^3 target FA image (FMRIB58_FA; FMRIB Software Library) using non-linear registration.[14,15] Aligned FA images were transformed into the Montreal Neurologic Institute 152 template using affine registrations. The study-specific mean FA image across all participants was calculated and thinned to create a study-specific mean FA skeleton which represents the centers of all tracts common to our sample. We applied a threshold

of 0.3 to the mean FA skeleton to form the final skeleton, a spatial representation of the majority of major white matter fiber bundles. Next, the values from the FA base templates of each participant were projected onto this skeleton by searching for the local center of the relevant fiber tract. In the final step, the aligned FA and MD image at T0 and T1 were projected onto the skeleton, creating four skeletons containing the aligned FA or MD values of every subject for both T0 and T1, to be used for the statistical analysis.

Statistical analysis

All skeletonized WM tracts described in the International Consortium of Brain Mapping (ICBM)-DTI-81 WM labels atlas [16,17] by John Hopkins University were quantified, except for the left and right tapetum. Mean FA and MD were calculated by averaging all voxels belonging to the skeletonized tract. In addition, we included the whole skeleton mean and mean value of all peripheral voxels, defined as voxels without ICBM-DTI-81 label, resulting in 48 'tracts'. Within each separate tract, extreme outliers defined as values 3 times the interquartile range above the third quartile or below the first quartile were determined using the 'rstatix' package[18] version 0.7.0 and excluded. As a result, sample sizes differ slightly per tract, and are reported in the result section. Tract-wise outcomes were analyzed using linear and linear mixed effect regression models using the LME4 package[19] version 1.1-30 in R[20] version 4.0.4. All p values were corrected for multiple testing using False Discovery Rate (FDR)-correction.[21] In addition to FDR-correction, we calculated a Bonferroni-based correction for multiple, correlated endpoints[22] (p_{corr}) given the between-tract correlation of FA values and of MD values. This method uses the interclass correlation to adjust for correlated measures, here tracts. The single fixed raters

interclass correlation coefficient (ICC) of MD (ICC_{MD} = 0.162) and FA (ICC_{FA} = 0.292) at week 0 (T0) was determined using the 'psych' package[23] version 2.2.5.

To assess the effect of baseline WM microstructure on improvements in insomnia and depressive symptom severity after treatment we utilized all follow-up measures of ISI and IDS-SR in a mixed effect analysis with age, sex and the baseline measure as covariate (e.g. ISI_{T1-4} ~ group + tract_FA_{T0} + group:tract_FA_{T0} + ISI_{T0} + age + sex) and a random intercept for each subject. Subsequently we combined CRS, CBT-I and CRS+CBT-I into a single 'intervention' group and repeated the analysis to capture general intervention by tract interaction effects on ISI and IDS-SR. Linear mixed-effect model p-values were estimated using the Satterthwaite's degrees of freedom method by the lmerTest package[24] version 3.1-3. In addition, we used the 'tidyverse' package[25] version 1.3.2 for data handling and visualization. The 'effects' package[26] version 4.2-2 was used to visualize and compute a linear regression of the interaction effects by predicting responses assuming averages over the other terms in the model (Figure 1A).

To assess the effect of insomnia interventions on WM microstructure, we used a linear regression model for each tract and tested whether the three active intervention groups differed significantly from the no treatment group at post-treatment (T1), while correcting for age, sex and corresponding baseline values (e.g. FA_{T1} ~ CBT-I + CRS + 'CBT-I+CRS' + age + sex + FA_{T0}). Reported standardized regression coefficients (β) were obtained by scaling all numeric variables before fitting the regression model.

Tract-wise analyses were complemented by voxel-wise analysis to investigate whether improvements in insomnia and depressive symptom severity after treatment were associated with baseline WM microstructure in specific parts of the tracts or more likely distributed across a tract or heterogeneous between subjects, i.e. not requiring sample convergence at the voxel level.[27]

Voxel-wise analyses of the FA and MD skeleton were carried out using the non-parametric permutation testing tool 'randomise'[28] within FSL. We tested whether the three active intervention groups differed significantly from the no treatment group at post-treatment (T1), while correcting for age, sex and corresponding baseline values (e.g. FA_{T1} ~ CBT-I + CRS + 'CBT-I+CRS' + age + sex + FA_{T0}). This was achieved by adding the demeaned baseline skeleton as voxel-wise regressor. For every analysis we ran 10.000 permutations and applied threshold-free cluster enhancement[29] (TFCE) with a significance level set at $p < .05$ and Family Wise Error correction for multiple comparisons. Age and sex were included as covariates.

References

1. Leerssen J, Foster-Dingley JC, Lakbila-Kamal O, et al. Internet-guided cognitive, behavioral and chronobiological interventions in depression-prone insomnia subtypes: Protocol of a randomized controlled prevention trial. *BMC Psychiatry*. 2020;20(1):1-11. doi:10/grzvxm

2. Leerssen J, Lakbila-Kamal O, Dekkers LMS, et al. Treating insomnia with high risk of depression using therapist-guided digital cognitive, behavioral, and circadian rhythm support interventions to prevent worsening of depressive symptoms: a randomized controlled trial. *Psychother Psychosom*. 2022;91(3):168-179. doi:10/gn9xww

3. Jack CR, Bernstein MA, Fox NC, et al. The Alzheimer's Disease Neuroimaging Initiative (ADNI): MRI methods. *J Magn Reson Imaging*. 2008;27(4):685-691. doi:10.1002/jmri.21049

4. Fischl B, Van Der Kouwe A, Destrieux C, et al. Automatically parcellating the human cerebral cortex. *Cerebr Cortex*. 2004;14(1):11-22. doi:10/dvg5ms

5. Lange SC de, Helwegen K, Heuvel MP van den. Structural and functional connectivity reconstruction with CATO - A Connectivity Analysis TOolbox. *NeuroImage*. 2023;273. doi:10/gscgvm

6. Andersson JLR, Sotiropoulos SN. An integrated approach to correction for off-resonance effects and subject movement in diffusion MR imaging. *NeuroImage*. 2016;125:1063-1078. doi:10.1016/j.neuroimage.2015.10.019

7. Andersson JLR, Graham MS, Zsoldos E, Sotiropoulos SN. Incorporating outlier detection and replacement into a non-parametric framework for movement and distortion correction of diffusion MR images. *NeuroImage*. 2016;141:556-572. doi:10.1016/j.neuroimage.2016.06.058

8. Smith SM, Jenkinson M, Woolrich MW, et al. Advances in functional and structural MR image analysis and implementation as FSL. *NeuroImage*. 2004;23:208-219. doi:10.1016/j.neuroimage.2004.07.051

9. Andersson JLR, Skare S, Ashburner J. How to correct susceptibility distortions in spin-echo echo-planar images: Application to diffusion tensor imaging. *NeuroImage*. 2003;20(2):870-888. doi:10.1016/S1053-8119(03)00336-7

10. Smith SM, Jenkinson M, Johansen-berg H, et al. Tract-based spatial statistics: Voxelwise analysis of multi-subject diffusion data. *NeuroImage*. 2006;31:1487-1505. doi:10/fnzkzd

11. Engvig A, Fjell AM, Westlye LT, et al. Memory training impacts short-term changes in aging white matter: A Longitudinal Diffusion Tensor Imaging Study. *Hum Brain Mapp*. 2012;33(10):2390-2406. doi:10/dmsv4q

12. Jenkinson M, Bannister P, Brady M, Smith S. Improved Optimization for the Robust and Accurate Linear Registration and Motion Correction of Brain Images. *NeuroImage*. 2002;17(2):825-841. doi:10.1006/nimg.2002.1132

13. Smith SM, De Stefano N, Jenkinson M, Matthews PM. Normalized accurate measurement of longitudinal brain change. *J Comput Assist Tomogr*. 2001;25(3):466-475. doi:10/bbpkbt

14. Andersson JLR, Jenkinson M, Smith S. Non-linear registration, aka spatial normalisation. FMRIB Technial Report TR07JA2. *www.fmrib.ox.ac.uk/analysis/techrep*. Published online 2007:1-21.

15. Andersson JLR, Jenkinson M, Smith SM. Non-linear optimisation. FMRIB technical report TR07JA1. *www.fmrib.ox.ac.uk/analysis/techrep*. Published online 2007:1-16.

16. Mori S, Oishi K, Jiang H, et al. Stereotaxic white matter atlas based on diffusion tensor imaging in an ICBM template. *NeuroImage*. 2008;40(2):570-582. doi:10/d35224

17. Susumu Mori, Lidia M. Nagae-Poetscher, Peter Van Zijl. *MRI Atlas of Human White Matter*. Elsevier; 2005.

18. Alboukadel Kassambara. Rstatix: pipe-friendly framework for basic statistical tests. Published online 2021.

19. Bates D, Maechler M, Bolker B, Walker S. Fitting linear mixed-effects models using lme4. *J Stat Softw*. 2015;67(1):1-48. doi:10/gcrnkw

20. R core team. R: A language and environment for statistical computing. *R Foundation for Statistical Computing, Vienna, Austria*. Published online 2017:R Foundation for Statistical Computing. doi:http://www.R-project.org/

21. Benjamini Y, Hochberg Y. Controlling the false discovery rate: a practical and powerful approach to multiple testing. *J R Statis Soc*. 1995;57(1):289-300. doi:10/gfpkdx

22. Shi Q, Pavey ES, Carter RE. Bonferroni-based correction factor for multiple, correlated endpoints. *Pharmaceut Statist*. 2012;11(4):300-309. doi:10/gscgvf

23. Revelle W. psych: Procedures for psychological, psychometric, and personality research. Published online 2022. https://CRAN.R-project.org/package=psych

24. Kuznetsova A, Brockhoff PB, Christensen RHB. lmerTest package: tests in linear mixed effects models. *J Stat Soft*. 2017;82(13). doi:10/dg3k

25. Wickham H, Averick M, Bryan J, et al. Welcome to the Tidyverse. *J Open Source Softw*. 2019;4(43):1686. doi:10.21105/joss.01686

26. Fox J. Effect Displays in R for Generalised Linear Models. *J Stat Softw*. 2003;8:1-27. doi:10.18637/jss.v008.i15

27. Van Someren EJW. Brain mechanisms of insomnia: new perspectives on causes and consequences. *Physiol Rev*. 2021;101(3):995-1046. doi:10/gmwnhj

28. Winkler AM, Ridgway GR, Webster MA, Smith SM, Nichols TE. Permutation inference for the general linear model. *NeuroImage*. 2014;92:381-397. doi:10.1016/j.neuroimage.2014.01.060

29. Smith SM, Nichols TE. Threshold-free cluster enhancement: Addressing problems of smoothing, threshold dependence and localisation in cluster inference. *NeuroImage*. 2009;44(1):83-98. doi:10.1016/j.neuroimage.2008.03.061

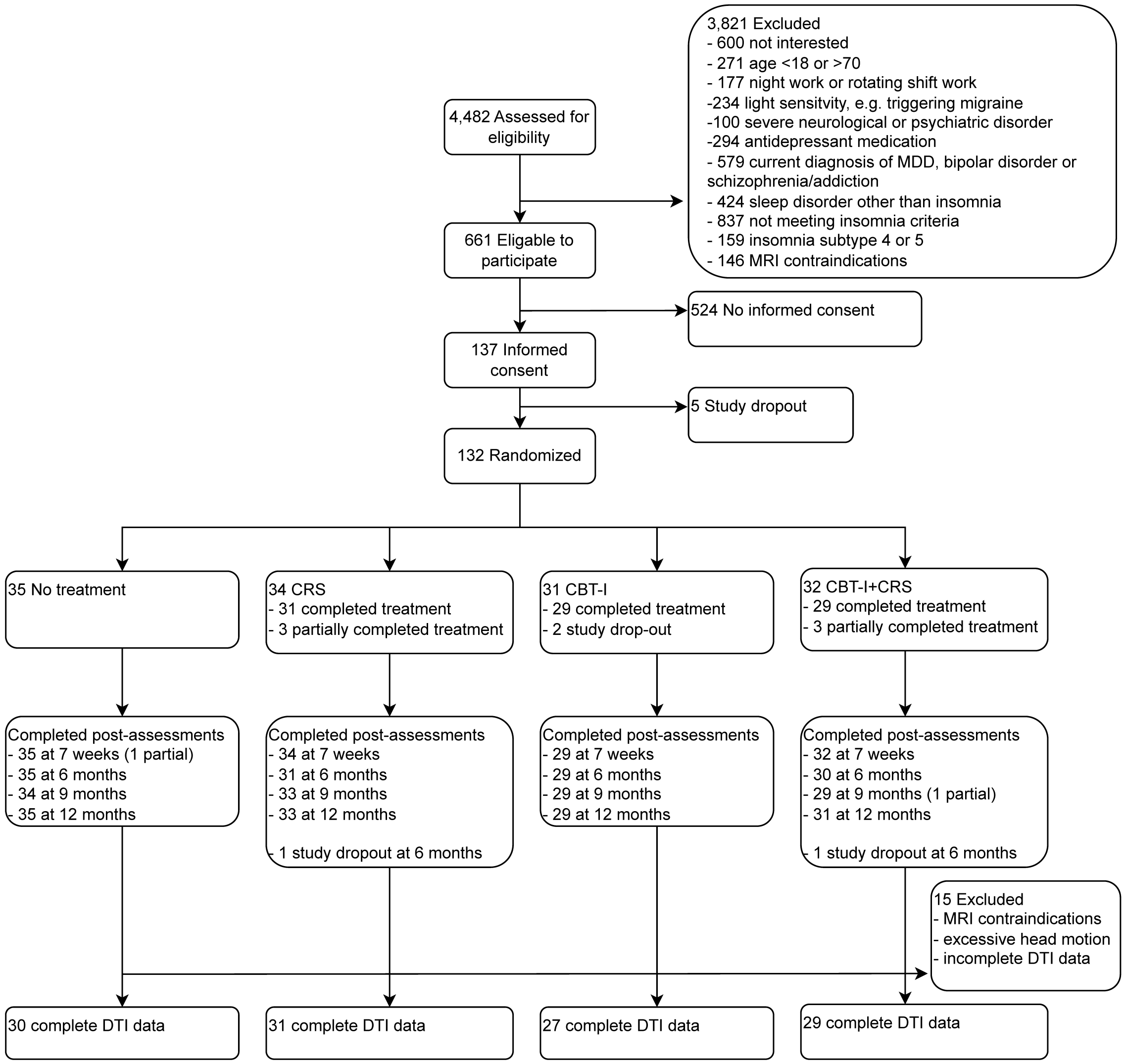

Supplementary figure 1. CONSORT flow diagram.
Flow of subjects at each stage in the randomized clinical trial. MDD, Major depressive disorder; CBT-I, Cognitive Behavioral Therapy for Insomnia; CRS, Circadian Rhythm Support; MRI, magnetic resonance imaging; DTI, diffusion tensor imaging; CONSORT, Consolidated Standards of Reporting Trials.

	CBT-I	CRS	CBT-I + CRS	NT	p
N	27	31	29	30	
Occupation [b]					0.010
- Employment	21 (77.8)	23 (74.2)	18 (62.1)	17 (56.7)	
- Incapacitated	1 (3.7)	0 (0.0)	1 (3.4)	3 (10.0)	
- Retired	4 (14.8)	5 (16.1)	3 (10.3)	2 (6.7)	
- Student	1 (3.7)	2 (6.5)	6 (20.7)	1 (3.3)	
- Unemployed	0 (0.0)	1 (3.2)	1 (3.4)	7 (23.3)	
Alcohol consumption per week [b]					0.698
- 0 units of alcohol	5 (18.5)	8 (25.8)	5 (17.2)	10 (33.3)	
- 1-14 units of alcohol	21 (77.8)	20 (64.5)	21 (72.4)	18 (60.0)	
- 15-21 units of alcohol	1 (3.7)	3 (9.7)	3 (10.3)	2 (6.7)	
Smoker [b]	1 (3.7)	2 (6.5)	1 (3.4)	2 (6.7)	0.910
Handedness [b]					0.161
- Right-handed	22 (81.5)	28 (90.3)	29 (100.0)	27 (90.0)	
- Left-handed	5 (18.5)	3 (9.7)	0 (0.0)	2 (6.7)	
- Both-handed	0 (0.0)	0 (0.0)	0 (0.0)	1 (3.3)	
Negative affect (PANAS) [a]	20.04 (7.10)	17.81 (4.70)	22.34 (6.28)	20.33 (5.79)	0.038
Positive affect (PANAS) [a]	31.41 (6.34)	30.90 (6.59)	31.97 (6.75)	30.53 (7.77)	0.868
Brain volume*	6.05 (0.04)	6.05 (0.04)	6.03 (0.05)	6.04 (0.05)	0.630
Total Gray matter Volume*	5.79 (0.04)	5.79 (0.04)	5.78 (0.04)	5.79 (0.05)	0.784
Cerebral White matter Volume*	5.67 (0.06)	5.67 (0.05)	5.65 (0.06)	5.67 (0.05)	0.586
mean FA	0.52 (0.02)	0.52 (0.02)	0.52 (0.01)	0.52 (0.02)	0.531
mean MD $\times 10^{-3}$	0.69 (0.03)	0.68 (0.02)	0.68 (0.02)	0.68 (0.02)	0.402

Supplementary table 1. Additional descriptive measures. [a] Mean (sd), [b] n (%) for each intervention group, * log10-scaled. CBT-I, cognitive behavioral therapy for insomnia; CRS, circadian rhythm support; NT, no treatment; PANAS, positive and negative affect schedule; FA, fractional anisotropy; MD, mean diffusivity.

	CBT-I	CRS	CBT-I + CRS	NT	p
N	27	31	29	30	
Antidepressants	0	0	1[a]	0	0.382
Anti-anxiety drugs	0	1	0	0	0.424
Anti-psychotics	0	0	0	0	-
Antihypertensives	4	0	1	5	0.051
Thyroid medication	0	2	1	0	0.325
Antiasthmatics	2	1	4	2	0.486
Anti-Parkinson drugs	0	0	0	0	-
Anticonvulsants	0	0	0	0	-
Headache medicines	3	1	1	0	0.207
Stimulants	0	1	1	0	0.585
Sleep medicines	5	4	2	5	0.592
Others	7	5	6	8	0.738

Supplementary table 2. Current use (i.e. past month) of medications for each intervention group (n). [a] low dose (12.5 mg) Quetiapine used as sleep medication. Given the low dosage and intended use, the participant was not excluded from analysis. CBT-I, cognitive behavioral therapy for insomnia; CRS, circadian rhythm support; NT, no treatment.

outcome	effect	tract	B	SE	t-value	p	p fdr	p corr
**ids-sr**	FA x combi	cerebral_peduncle_l	181.94	56.35	3.23	0.002	0.078	0.055
	FA x CBT-I	retrolenticular_part_of_internal_capsule_l	148.94	47.69	3.12	0.002	0.109	0.078
	FA x CRS	retrolenticular_part_of_internal_capsule_l	160.47	53.63	2.99	0.003	0.163	0.116
	FA x combi	retrolenticular_part_of_internal_capsule_l	144.92	52.05	2.78	0.006	0.150	0.215
	FA x CBT-I	anterior_corona_radiata_r	133.70	48.60	2.75	0.007	0.166	0.237
	FA x combi	superior_cerebellar_peduncle_l	153.83	62.16	2.47	0.015	0.236	0.506
	FA x CRS	anterior_corona_radiata_r	125.20	51.10	2.45	0.016	0.380	0.542
	FA x CBT-I	fornix_cres_stria_terminalis_can_not_be_resolved_with_current_resolution_l	121.43	49.99	2.43	0.017	0.267	0.573
	FA x combi	splenium_of_corpus_callosum	36.27	16.17	2.24	0.027	0.259	0.921
	FA x combi	cerebral_peduncle_r	117.25	53.05	2.21	0.029	0.259	0.997
	FA x combi	anterior_limb_of_internal_capsule_r	131.15	60.56	2.17	0.032	0.259	1.000
	FA x CBT-I	cerebral_peduncle_l	120.20	58.26	2.06	0.041	0.301	1.000
	FA x combi	anterior_corona_radiata_r	112.30	54.71	2.05	0.042	0.279	1.000
	FA x CBT-I	superior_longitudinal_fasciculus_r	108.78	53.83	2.02	0.046	0.301	1.000
	FA x CBT-I	splenium_of_corpus_callosum	32.00	15.98	2.00	0.048	0.301	1.000
	FA x CBT-I	inferior_cerebellar_peduncle_r	-144.80	72.90	-1.99	0.049	0.301	1.000
	FA x combi	middle_cerebellar_peduncle	75.42	38.22	1.97	0.051	0.279	1.000
	FA x combi	mean_skel	173.22	88.31	1.96	0.052	0.279	1.000
	FA x CBT-I	superior_corona_radiata_r	97.92	50.63	1.93	0.056	0.301	1.000
	FA x CBT-I	medial_lemniscus_l	-128.09	66.46	-1.93	0.056	0.301	1.000
	FA x combi	retrolenticular_part_of_internal_capsule_r	86.67	46.41	1.87	0.064	0.300	1.000
	FA x combi	posterior_limb_of_internal_capsule_l	84.22	46.25	1.82	0.071	0.300	1.000
	FA x CRS	splenium_of_corpus_callosum	30.10	16.59	1.81	0.072	0.879	1.000
	FA x combi	pontine_crossing_tract_a_part_of_mcp	110.88	61.71	1.80	0.075	0.300	1.000
	FA x CBT-I	posterior_limb_of_internal_capsule_l	77.44	43.13	1.80	0.075	0.360	1.000
	FA x CBT-I	retrolenticular_part_of_internal_capsule_r	75.27	42.97	1.75	0.083	0.360	1.000
	FA x CRS	fornix_cres_stria_terminalis_can_not_be_resolved_with_current_resolution_l	103.36	59.62	1.73	0.086	0.879	1.000
	FA x combi	posterior_corona_radiata_r	110.22	63.70	1.73	0.086	0.303	1.000
	FA x combi	posterior_limb_of_internal_capsule_r	98.77	57.48	1.72	0.088	0.303	1.000
	FA x CBT-I	anterior_limb_of_internal_capsule_r	106.23	62.11	1.71	0.090	0.360	1.000
	FA x combi	no_label_skel	122.26	73.74	1.66	0.100	0.320	1.000
	FA x CRS	medial_lemniscus_l	-109.99	67.17	-1.64	0.104	0.879	1.000
	FA x CBT-I	sagittal_stratum_include_inferior_longitidinal_fasciculus_and_inferior_fronto_minus_occipital_fasciculus_r	72.98	46.66	1.56	0.121	0.406	1.000
	FA x combi	body_of_corpus_callosum	101.67	65.22	1.56	0.122	0.360	1.000
	FA x CBT-I	mean_skel	124.16	80.53	1.54	0.126	0.406	1.000
	FA x CBT-I	cingulum_cingulate_gyrus_r	69.81	45.38	1.54	0.127	0.406	1.000
	FA x combi	superior_corona_radiata_r	81.30	52.94	1.54	0.127	0.360	1.000
	FA x combi	genu_of_corpus_callosum	93.54	62.79	1.49	0.139	0.362	1.000
	FA x CRS	inferior_cerebellar_peduncle_r	-110.03	74.30	-1.48	0.141	0.879	1.000
	FA x combi	superior_longitudinal_fasciculus_l	73.26	49.73	1.47	0.143	0.362	1.000
	FA x CRS	retrolenticular_part_of_internal_capsule_r	81.37	57.32	1.42	0.158	0.879	1.000
	FA x combi	corticospinal_tract_r	60.09	43.47	1.38	0.170	0.389	1.000
	FA x combi	cingulum_hippocampus_l	66.18	48.32	1.37	0.173	0.389	1.000
	FA x combi	posterior_thalamic_radiation_include_optic_radiation_l	57.06	43.51	1.31	0.192	0.389	1.000
	FA x CBT-I	pontine_crossing_tract_a_part_of_mcp	73.25	56.04	1.31	0.194	0.564	1.000
	FA x combi	posterior_corona_radiata_l	64.20	49.45	1.30	0.197	0.389	1.000
	FA x CBT-I	posterior_thalamic_radiation_include_optic_radiation_l	54.02	41.87	1.29	0.200	0.564	1.000
	FA x combi	inferior_cerebellar_peduncle_r	-99.17	77.88	-1.27	0.205	0.389	1.000
	FA x combi	posterior_thalamic_radiation_include_optic_radiation_r	59.97	48.22	1.24	0.216	0.389	1.000
	FA x CRS	mean_skel	120.80	97.13	1.24	0.216	0.879	1.000
	FA x combi	fornix_cres_stria_terminalis_can_not_be_resolved_with_current_resolution_l	73.57	59.36	1.24	0.218	0.389	1.000
	FA x CRS	cingulum_cingulate_gyrus_l	-48.13	39.41	-1.22	0.224	0.879	1.000
	FA x combi	cingulum_cingulate_gyrus_r	55.87	45.81	1.22	0.225	0.389	1.000

FA x combi	fornix_cres_stria_terminalis_can_not_be_resolved_with_current_resolution_r	54.21	44.62	1.21	0.227	0.389	1.000
FA x CBT-I	anterior_corona_radiata_l	61.67	51.46	1.20	0.233	0.620	1.000
FA x CRS	superior_longitudinal_fasciculus_r	73.42	62.91	1.17	0.246	0.879	1.000
FA x CBT-I	cingulum_hippocampus_r	36.82	31.69	1.16	0.248	0.620	1.000
FA x CRS	uncinate_fasciculus_l	-41.00	35.50	-1.15	0.251	0.879	1.000
FA x combi	inferior_cerebellar_peduncle_l	62.68	54.29	1.15	0.251	0.415	1.000
FA x CBT-I	uncinate_fasciculus_r	-27.85	24.80	-1.12	0.264	0.620	1.000
FA x CRS	inferior_cerebellar_peduncle_l	63.72	56.97	1.12	0.266	0.879	1.000
FA x CBT-I	cerebral_peduncle_r	56.92	51.50	1.11	0.271	0.620	1.000
FA x combi	fornix_column_and_body_of_fornix	52.30	50.15	1.04	0.299	0.479	1.000
FA x CRS	cingulum_cingulate_gyrus_r	52.12	51.35	1.02	0.312	0.879	1.000
FA x CRS	anterior_limb_of_internal_capsule_r	51.40	53.31	0.96	0.337	0.879	1.000
FA x combi	anterior_corona_radiata_l	59.01	61.49	0.96	0.339	0.512	1.000
FA x combi	cingulum_hippocampus_r	29.85	31.59	0.94	0.347	0.512	1.000
FA x CBT-I	corticospinal_tract_l	-64.56	68.85	-0.94	0.350	0.717	1.000
FA x CRS	body_of_corpus_callosum	61.96	66.10	0.94	0.350	0.879	1.000
FA x CBT-I	posterior_limb_of_internal_capsule_r	55.41	59.39	0.93	0.353	0.717	1.000
FA x combi	sagittal_stratum_include_inferior_longitidinal_fasciculus_and_inferior_fronto_minus_occipital_fasciculus_l	61.61	66.76	0.92	0.358	0.512	1.000
FA x combi	superior_longitudinal_fasciculus_r	57.60	63.01	0.91	0.363	0.512	1.000
FA x CRS	cerebral_peduncle_l	50.68	56.08	0.90	0.368	0.879	1.000
FA x CBT-I	posterior_thalamic_radiation_include_optic_radiation_r	38.15	42.22	0.90	0.368	0.717	1.000
FA x CBT-I	inferior_cerebellar_peduncle_l	53.44	59.77	0.89	0.373	0.717	1.000
FA x CRS	pontine_crossing_tract_a_part_of_mcp	56.00	63.97	0.88	0.383	0.879	1.000
FA x combi	medial_lemniscus_l	-59.54	68.56	-0.87	0.387	0.531	1.000
FA x CRS	posterior_thalamic_radiation_include_optic_radiation_l	40.71	47.49	0.86	0.393	0.879	1.000
FA x CRS	superior_corona_radiata_r	43.19	50.64	0.85	0.396	0.879	1.000
FA x CRS	anterior_corona_radiata_l	48.52	59.86	0.81	0.419	0.879	1.000
FA x CRS	posterior_limb_of_internal_capsule_l	36.92	45.61	0.81	0.420	0.879	1.000
FA x CBT-I	uncinate_fasciculus_l	-26.52	33.03	-0.80	0.424	0.746	1.000
FA x CBT-I	superior_fronto_minus_occipital_fasciculus_could_be_a_part_of_anterior_internal_capsule_r	34.83	43.59	0.80	0.426	0.746	1.000
FA x CRS	fornix_column_and_body_of_fornix	40.29	51.31	0.79	0.434	0.879	1.000
FA x CBT-I	superior_cerebellar_peduncle_r	46.30	59.27	0.78	0.436	0.746	1.000
FA x CRS	middle_cerebellar_peduncle	37.18	47.83	0.78	0.439	0.879	1.000
FA x CBT-I	external_capsule_l	34.65	46.02	0.75	0.453	0.746	1.000
FA x CRS	external_capsule_l	39.10	52.72	0.74	0.460	0.879	1.000
FA x CRS	posterior_corona_radiata_l	38.49	52.54	0.73	0.465	0.879	1.000
FA x CBT-I	fornix_cres_stria_terminalis_can_not_be_resolved_with_current_resolution_r	30.98	42.38	0.73	0.466	0.746	1.000
FA x combi	uncinate_fasciculus_l	25.84	35.81	0.72	0.472	0.622	1.000
FA x combi	superior_fronto_minus_occipital_fasciculus_could_be_a_part_of_anterior_internal_capsule_r	29.54	41.65	0.71	0.480	0.622	1.000
FA x CBT-I	superior_longitudinal_fasciculus_l	33.92	48.35	0.70	0.484	0.746	1.000
FA x CBT-I	superior_fronto_minus_occipital_fasciculus_could_be_a_part_of_anterior_internal_capsule_l	-22.06	32.38	-0.68	0.497	0.746	1.000
FA x CRS	sagittal_stratum_include_inferior_longitidinal_fasciculus_and_inferior_fronto_minus_occipital_fasciculus_r	33.28	50.29	0.66	0.509	0.879	1.000
FA x CRS	no_label_skel	47.16	71.42	0.66	0.510	0.879	1.000
FA x CRS	corticospinal_tract_r	28.16	42.87	0.66	0.513	0.879	1.000
FA x combi	anterior_limb_of_internal_capsule_l	37.20	58.28	0.64	0.525	0.663	1.000
FA x combi	superior_cerebellar_peduncle_r	33.41	55.82	0.60	0.551	0.678	1.000
FA x CBT-I	corticospinal_tract_r	25.18	42.07	0.60	0.551	0.801	1.000

	FA x CBT-I	sagittal_stratum_include_inferior_longitidinal_fasciculus_and_inferior_fronto_minus_occipital_fasciculus_l	28.16	52.01	0.54	0.589	0.808	1.000
	FA x CRS	anterior_limb_of_internal_capsule_l	-35.52	67.50	-0.53	0.600	0.928	1.000
	FA x CBT-I	genu_of_corpus_callosum	29.50	56.44	0.52	0.602	0.808	1.000
	FA x CBT-I	external_capsule_r	-26.59	52.90	-0.50	0.616	0.808	1.000
	FA x CRS	superior_fronto_minus_occipital_fasciculus_could_be_a_part_of_anterior_internal_capsule_l	-18.12	36.12	-0.50	0.617	0.928	1.000
	FA x CBT-I	middle_cerebellar_peduncle	19.11	38.78	0.49	0.623	0.808	1.000
	FA x CRS	superior_cerebellar_peduncle_r	28.50	58.12	0.49	0.625	0.928	1.000
	FA x CRS	posterior_thalamic_radiation_include_optic_radiation_r	24.19	50.79	0.48	0.635	0.928	1.000
	FA x CRS	cerebral_peduncle_r	26.73	61.04	0.44	0.662	0.928	1.000
	FA x CRS	medial_lemniscus_r	25.87	62.15	0.42	0.678	0.928	1.000
	FA x CBT-I	anterior_limb_of_internal_capsule_l	-23.42	56.78	-0.41	0.681	0.852	1.000
	FA x combi	external_capsule_l	20.32	49.65	0.41	0.683	0.790	1.000
	FA x CRS	corticospinal_tract_l	-29.81	72.94	-0.41	0.684	0.928	1.000
	FA x CBT-I	superior_cerebellar_peduncle_l	21.63	54.55	0.40	0.692	0.852	1.000
	FA x combi	medial_lemniscus_r	26.19	66.86	0.39	0.696	0.790	1.000
	FA x combi	cingulum_cingulate_gyrus_l	15.65	42.07	0.37	0.711	0.790	1.000
	FA x CRS	posterior_limb_of_internal_capsule_r	24.90	68.69	0.36	0.718	0.928	1.000
	FA x combi	superior_corona_radiata_l	-21.68	60.56	-0.36	0.721	0.790	1.000
	FA x combi	superior_fronto_minus_occipital_fasciculus_could_be_a_part_of_anterior_internal_capsule_l	11.30	31.98	0.35	0.725	0.790	1.000
	FA x CRS	fornix_cres_stria_terminalis_can_not_be_resolved_with_current_resolution_r	13.87	42.34	0.33	0.744	0.928	1.000
	FA x CRS	uncinate_fasciculus_r	-9.02	28.26	-0.32	0.750	0.928	1.000
	FA x CRS	superior_cerebellar_peduncle_l	-20.42	65.76	-0.31	0.757	0.928	1.000
	FA x combi	sagittal_stratum_include_inferior_longitidinal_fasciculus_and_inferior_fronto_minus_occipital_fasciculus_r	13.21	45.68	0.29	0.773	0.824	1.000
	FA x CRS	sagittal_stratum_include_inferior_longitidinal_fasciculus_and_inferior_fronto_minus_occipital_fasciculus_l	-16.35	62.38	-0.26	0.794	0.928	1.000
	FA x CRS	superior_fronto_minus_occipital_fasciculus_could_be_a_part_of_anterior_internal_capsule_r	10.64	41.08	0.26	0.796	0.928	1.000
	FA x CBT-I	fornix_column_and_body_of_fornix	11.91	47.12	0.25	0.801	0.959	1.000
	FA x CRS	genu_of_corpus_callosum	-13.34	58.87	-0.23	0.821	0.928	1.000
	FA x combi	corticospinal_tract_l	14.26	68.60	0.21	0.836	0.872	1.000
	FA x CRS	superior_corona_radiata_l	-12.50	61.75	-0.20	0.840	0.928	1.000
	FA x CBT-I	posterior_corona_radiata_l	9.94	49.16	0.20	0.840	0.959	1.000
	FA x CRS	posterior_corona_radiata_r	-9.01	47.60	-0.19	0.850	0.928	1.000
	FA x CBT-I	medial_lemniscus_r	-8.93	64.19	-0.14	0.890	0.959	1.000
	FA x CRS	superior_longitudinal_fasciculus_l	6.76	50.83	0.13	0.894	0.954	1.000
	FA x CBT-I	posterior_corona_radiata_r	6.59	53.86	0.12	0.903	0.959	1.000
	FA x combi	uncinate_fasciculus_r	2.92	26.34	0.11	0.912	0.931	1.000
	FA x CBT-I	body_of_corpus_callosum	7.17	65.37	0.11	0.913	0.959	1.000
	FA x CBT-I	cingulum_cingulate_gyrus_l	-4.19	39.19	-0.11	0.915	0.959	1.000
	FA x CBT-I	superior_corona_radiata_l	-4.47	51.65	-0.09	0.931	0.959	1.000
	FA x CBT-I	no_label_skel	-5.94	77.75	-0.08	0.939	0.959	1.000
	FA x CRS	cingulum_hippocampus_r	1.91	33.02	0.06	0.954	0.968	1.000
	FA x CRS	cingulum_hippocampus_l	-2.17	47.90	-0.05	0.964	0.968	1.000
	FA x CRS	external_capsule_r	2.40	59.88	0.04	0.968	0.968	1.000
	FA x CBT-I	cingulum_hippocampus_l	1.57	47.25	0.03	0.974	0.974	1.000
	FA x combi	external_capsule_r	-1.22	59.76	-0.02	0.984	0.984	1.000
**isi**	FA x combi	middle_cerebellar_peduncle	48.20	18.99	2.54	0.012	0.433	0.425
	FA x CRS	posterior_corona_radiata_l	-70.37	28.13	-2.50	0.014	0.264	0.471
	FA x CRS	inferior_cerebellar_peduncle_l	72.20	29.19	2.47	0.015	0.264	0.504
	FA x CRS	anterior_limb_of_internal_capsule_l	-87.34	36.02	-2.42	0.017	0.264	0.578

FA x CRS	superior_cerebellar_peduncle_r	67.72	29.20	2.32	0.022	0.264	0.755
FA x combi	inferior_cerebellar_peduncle_r	-95.28	41.27	-2.31	0.023	0.433	0.778
FA x combi	inferior_cerebellar_peduncle_l	61.64	27.54	2.24	0.027	0.433	0.928
FA x CRS	posterior_limb_of_internal_capsule_r	-76.92	36.92	-2.08	0.039	0.378	1.000
FA x CBT-I	fornix_cres_stria_terminalis_can_not_be_resolved_with_current_resolution_r	-46.56	22.82	-2.04	0.044	0.704	1.000
FA x CRS	posterior_thalamic_radiation_include_optic_radiation_r	-53.96	27.02	-2.00	0.048	0.386	1.000
FA x CBT-I	external_capsule_r	-55.06	28.23	-1.95	0.054	0.704	1.000
FA x CBT-I	posterior_limb_of_internal_capsule_r	-60.74	31.51	-1.93	0.056	0.704	1.000
FA x combi	sagittal_stratum_include_inferior_longitidinal_fasciculus_and_inferior_fronto_minus_occipital_fasciculus_r	-45.97	24.07	-1.91	0.059	0.704	1.000
FA x CBT-I	superior_cerebellar_peduncle_r	55.83	29.57	1.89	0.062	0.704	1.000
FA x CRS	uncinate_fasciculus_l	-35.13	19.12	-1.84	0.069	0.471	1.000
FA x CRS	middle_cerebellar_peduncle	41.72	23.71	1.76	0.081	0.487	1.000
FA x combi	superior_cerebellar_peduncle_r	47.93	27.69	1.73	0.086	0.826	1.000
FA x CBT-I	uncinate_fasciculus_l	-30.37	17.86	-1.70	0.092	0.704	1.000
FA x combi	medial_lemniscus_l	-59.58	37.07	-1.61	0.111	0.885	1.000
FA x CBT-I	inferior_cerebellar_peduncle_l	46.15	30.46	1.52	0.133	0.704	1.000
FA x CBT-I	anterior_limb_of_internal_capsule_r	-50.18	33.77	-1.49	0.140	0.704	1.000
FA x CBT-I	anterior_limb_of_internal_capsule_l	-44.67	30.36	-1.47	0.144	0.704	1.000
FA x CBT-I	superior_longitudinal_fasciculus_l	-37.15	26.04	-1.43	0.157	0.704	1.000
FA x CBT-I	sagittal_stratum_include_inferior_longitidinal_fasciculus_and_inferior_fronto_minus_occipital_fasciculus_l	-37.55	28.08	-1.34	0.184	0.704	1.000
FA x CRS	corticospinal_tract_r	28.94	21.76	1.33	0.186	0.810	1.000
FA x CBT-I	sagittal_stratum_include_inferior_longitidinal_fasciculus_and_inferior_fronto_minus_occipital_fasciculus_r	-32.25	25.21	-1.28	0.203	0.704	1.000
FA x CBT-I	uncinate_fasciculus_r	-17.21	13.45	-1.28	0.203	0.704	1.000
FA x combi	posterior_limb_of_internal_capsule_r	-39.16	30.76	-1.27	0.206	0.902	1.000
FA x CBT-I	mean_skel	-54.13	43.54	-1.24	0.216	0.704	1.000
FA x combi	posterior_thalamic_radiation_include_optic_radiation_l	-29.45	23.83	-1.24	0.219	0.902	1.000
FA x CBT-I	posterior_corona_radiata_r	-35.93	29.33	-1.23	0.223	0.704	1.000
FA x combi	fornix_column_and_body_of_fornix	29.04	24.05	1.21	0.230	0.902	1.000
FA x CBT-I	superior_corona_radiata_l	-33.98	28.18	-1.21	0.230	0.704	1.000
FA x combi	superior_corona_radiata_l	-37.82	32.55	-1.16	0.248	0.902	1.000
FA x CBT-I	anterior_corona_radiata_l	-32.05	28.27	-1.13	0.259	0.704	1.000
FA x CRS	fornix_column_and_body_of_fornix	28.03	25.05	1.12	0.265	0.810	1.000
FA x combi	anterior_corona_radiata_l	-36.30	33.05	-1.10	0.274	0.902	1.000
FA x CBT-I	posterior_corona_radiata_l	-28.48	25.96	-1.10	0.275	0.704	1.000
FA x CBT-I	corticospinal_tract_r	-22.96	21.21	-1.08	0.281	0.704	1.000
FA x CRS	superior_corona_radiata_l	-35.81	33.24	-1.08	0.284	0.810	1.000
FA x combi	retrolenticular_part_of_internal_capsule_r	-27.53	25.65	-1.07	0.285	0.902	1.000
FA x combi	anterior_limb_of_internal_capsule_l	-33.24	31.11	-1.07	0.287	0.902	1.000
FA x CRS	fornix_cres_stria_terminalis_can_not_be_resolved_with_current_resolution_l	35.99	33.74	1.07	0.288	0.810	1.000
FA x combi	uncinate_fasciculus_l	-20.59	19.32	-1.07	0.289	0.902	1.000
FA x CBT-I	anterior_corona_radiata_r	-27.89	26.29	-1.06	0.291	0.704	1.000
FA x CBT-I	pontine_crossing_tract_a_part_of_mcp	-30.57	28.95	-1.06	0.293	0.704	1.000
FA x CRS	superior_cerebellar_peduncle_l	-38.48	36.98	-1.04	0.300	0.810	1.000
FA x combi	cingulum_hippocampus_l	26.66	26.13	1.02	0.310	0.902	1.000
FA x CRS	anterior_limb_of_internal_capsule_r	-29.61	29.45	-1.01	0.317	0.810	1.000
FA x CBT-I	cerebral_peduncle_r	-27.78	28.44	-0.98	0.331	0.756	1.000
FA x CRS	medial_lemniscus_l	-35.30	36.26	-0.97	0.332	0.810	1.000
FA x CRS	cingulum_hippocampus_l	-25.31	26.06	-0.97	0.334	0.810	1.000
FA x CRS	posterior_thalamic_radiation_include_optic_radiation_l	-24.84	26.11	-0.95	0.343	0.810	1.000
FA x CRS	pontine_crossing_tract_a_part_of_mcp	-31.87	33.90	-0.94	0.349	0.810	1.000
FA x combi	body_of_corpus_callosum	33.55	35.88	0.94	0.352	0.902	1.000

FA x combi	superior_fronto_minus_occipital_fasciculus_could_be_a_part_of_anterior_internal_capsule_l	16.42	17.73	0.93	0.356	0.902	1.000
FA x combi	cerebral_peduncle_l	29.15	31.61	0.92	0.358	0.902	1.000
FA x CRS	mean_skel	-48.07	52.66	-0.91	0.363	0.810	1.000
FA x combi	superior_cerebellar_peduncle_l	31.29	34.37	0.91	0.364	0.902	1.000
FA x CRS	genu_of_corpus_callosum	-28.52	31.61	-0.90	0.369	0.810	1.000
FA x CRS	cerebral_peduncle_r	-31.04	34.53	-0.90	0.371	0.810	1.000
FA x CRS	anterior_corona_radiata_r	-25.06	27.91	-0.90	0.371	0.810	1.000
FA x CRS	superior_fronto_minus_occipital_fasciculus_could_be_a_part_of_anterior_internal_capsule_r	-19.00	22.12	-0.86	0.392	0.818	1.000
FA x combi	medial_lemniscus_r	-30.24	36.04	-0.84	0.403	0.902	1.000
FA x CBT-I	fornix_cres_stria_terminalis_can_not_be_resolved_with_current_resolution_l	23.32	28.57	0.82	0.416	0.833	1.000
FA x combi	posterior_corona_radiata_r	27.97	34.72	0.81	0.422	0.902	1.000
FA x combi	splenium_of_corpus_callosum	6.91	8.84	0.78	0.436	0.902	1.000
FA x CRS	fornix_cres_stria_terminalis_can_not_be_resolved_with_current_resolution_r	-17.47	22.53	-0.78	0.440	0.854	1.000
FA x CRS	inferior_cerebellar_peduncle_r	-30.08	39.23	-0.77	0.445	0.854	1.000
FA x CBT-I	cerebral_peduncle_l	-24.46	32.70	-0.75	0.456	0.833	1.000
FA x CBT-I	retrolenticular_part_of_internal_capsule_l	-19.23	26.50	-0.73	0.469	0.833	1.000
FA x CRS	cerebral_peduncle_l	-22.61	31.66	-0.71	0.476	0.880	1.000
FA x CBT-I	superior_fronto_minus_occipital_fasciculus_could_be_a_part_of_anterior_internal_capsule_r	-16.53	23.37	-0.71	0.481	0.833	1.000
FA x CBT-I	posterior_thalamic_radiation_include_optic_radiation_r	-15.40	22.21	-0.69	0.489	0.833	1.000
FA x combi	anterior_corona_radiata_r	-19.57	29.70	-0.66	0.511	0.902	1.000
FA x combi	external_capsule_r	-20.93	31.95	-0.66	0.514	0.902	1.000
FA x combi	posterior_corona_radiata_l	-17.08	26.27	-0.65	0.517	0.902	1.000
FA x combi	uncinate_fasciculus_r	9.06	14.18	0.64	0.524	0.902	1.000
FA x CRS	cingulum_cingulate_gyrus_l	-13.86	21.76	-0.64	0.525	0.915	1.000
FA x CBT-I	middle_cerebellar_peduncle	11.96	19.06	0.63	0.531	0.833	1.000
FA x CBT-I	genu_of_corpus_callosum	-18.24	30.43	-0.60	0.550	0.833	1.000
FA x combi	corticospinal_tract_r	12.78	21.93	0.58	0.561	0.902	1.000
FA x CRS	superior_longitudinal_fasciculus_r	20.10	34.84	0.58	0.565	0.915	1.000
FA x CBT-I	posterior_thalamic_radiation_include_optic_radiation_l	-13.13	23.01	-0.57	0.570	0.833	1.000
FA x CBT-I	retrolenticular_part_of_internal_capsule_r	-13.19	23.87	-0.55	0.582	0.833	1.000
FA x CRS	no_label_skel	-21.39	38.95	-0.55	0.584	0.915	1.000
FA x CBT-I	no_label_skel	22.88	41.72	0.55	0.584	0.833	1.000
FA x CRS	medial_lemniscus_r	17.89	33.67	0.53	0.596	0.915	1.000
FA x CBT-I	splenium_of_corpus_callosum	4.59	8.76	0.52	0.601	0.833	1.000
FA x combi	fornix_cres_stria_terminalis_can_not_be_resolved_with_current_resolution_r	-12.48	23.82	-0.52	0.602	0.902	1.000
FA x CBT-I	external_capsule_l	-12.39	24.76	-0.50	0.618	0.833	1.000
FA x combi	cingulum_hippocampus_r	-8.36	17.42	-0.48	0.632	0.902	1.000
FA x CBT-I	cingulum_hippocampus_l	-12.20	25.85	-0.47	0.638	0.833	1.000
FA x CBT-I	medial_lemniscus_r	-16.20	34.57	-0.47	0.640	0.833	1.000
FA x CBT-I	corticospinal_tract_l	-16.79	37.68	-0.45	0.657	0.833	1.000
FA x CBT-I	body_of_corpus_callosum	15.76	35.71	0.44	0.660	0.833	1.000
FA x combi	cerebral_peduncle_r	12.90	29.27	0.44	0.660	0.902	1.000
FA x CRS	posterior_limb_of_internal_capsule_l	-11.18	25.41	-0.44	0.661	0.915	1.000
FA x CBT-I	superior_corona_radiata_r	-11.94	28.45	-0.42	0.676	0.833	1.000
FA x combi	cingulum_cingulate_gyrus_r	10.37	24.74	0.42	0.676	0.902	1.000
FA x CBT-I	medial_lemniscus_l	-14.50	35.64	-0.41	0.685	0.833	1.000
FA x combi	external_capsule_l	-10.49	26.89	-0.39	0.697	0.902	1.000
FA x CRS	cingulum_cingulate_gyrus_r	10.77	27.76	0.39	0.699	0.915	1.000
FA x combi	mean_skel	-18.53	47.73	-0.39	0.699	0.902	1.000
FA x CBT-I	fornix_column_and_body_of_fornix	-8.20	22.17	-0.37	0.712	0.833	1.000
FA x combi	cingulum_cingulate_gyrus_l	-8.49	23.35	-0.36	0.717	0.902	1.000

FA x combi	posterior_thalamic_radiation_include_optic_radiation_r	-9.22	25.53	-0.36	0.719	0.902	1.000
FA x combi	corticospinal_tract_l	-13.51	37.55	-0.36	0.720	0.902	1.000
FA x CBT-I	superior_fronto_minus_occipital_fasciculus_could_be_a_part_of_anterior_internal_capsule_l	6.35	17.88	0.36	0.723	0.833	1.000
FA x CRS	corticospinal_tract_l	14.02	40.07	0.35	0.727	0.915	1.000
FA x CBT-I	cingulum_cingulate_gyrus_l	-7.61	21.90	-0.35	0.729	0.833	1.000
FA x combi	genu_of_corpus_callosum	11.49	33.81	0.34	0.735	0.902	1.000
FA x combi	anterior_limb_of_internal_capsule_r	11.14	33.06	0.34	0.737	0.902	1.000
FA x CRS	sagittal_stratum_include_inferior_longitidinal_fasciculus_and_inferior_fronto_minus_occipital_fasciculus_l	10.57	33.60	0.31	0.754	0.915	1.000
FA x CRS	superior_corona_radiata_r	-8.71	28.22	-0.31	0.758	0.915	1.000
FA x CRS	superior_longitudinal_fasciculus_l	-8.32	27.05	-0.31	0.759	0.915	1.000
FA x CRS	posterior_corona_radiata_r	-7.91	26.22	-0.30	0.764	0.915	1.000
FA x CRS	superior_fronto_minus_occipital_fasciculus_could_be_a_part_of_anterior_internal_capsule_l	-5.95	19.91	-0.30	0.766	0.915	1.000
FA x combi	sagittal_stratum_include_inferior_longitidinal_fasciculus_and_inferior_fronto_minus_occipital_fasciculus_l	10.68	35.86	0.30	0.766	0.902	1.000
FA x combi	superior_longitudinal_fasciculus_l	-7.40	26.44	-0.28	0.780	0.902	1.000
FA x CRS	uncinate_fasciculus_r	-4.11	15.45	-0.27	0.791	0.915	1.000
FA x combi	retrolenticular_part_of_internal_capsule_l	-7.48	28.91	-0.26	0.796	0.902	1.000
FA x combi	superior_corona_radiata_r	7.56	29.54	0.26	0.799	0.902	1.000
FA x CBT-I	superior_longitudinal_fasciculus_r	7.61	29.97	0.25	0.800	0.893	1.000
FA x combi	posterior_limb_of_internal_capsule_l	-6.25	25.67	-0.24	0.808	0.902	1.000
FA x CRS	splenium_of_corpus_callosum	2.22	9.16	0.24	0.809	0.915	1.000
FA x CRS	retrolenticular_part_of_internal_capsule_r	-7.60	31.88	-0.24	0.812	0.915	1.000
FA x CRS	sagittal_stratum_include_inferior_longitidinal_fasciculus_and_inferior_fronto_minus_occipital_fasciculus_r	-6.29	27.08	-0.23	0.817	0.915	1.000
FA x CRS	retrolenticular_part_of_internal_capsule_l	-6.86	30.04	-0.23	0.820	0.915	1.000
FA x CRS	cingulum_hippocampus_r	2.42	18.21	0.13	0.894	0.974	1.000
FA x combi	superior_fronto_minus_occipital_fasciculus_could_be_a_part_of_anterior_internal_capsule_r	2.79	22.44	0.12	0.901	0.967	1.000
FA x combi	no_label_skel	4.74	40.24	0.12	0.906	0.967	1.000
FA x CBT-I	superior_cerebellar_peduncle_l	-3.43	30.26	-0.11	0.910	0.963	1.000
FA x CRS	body_of_corpus_callosum	-4.02	36.58	-0.11	0.913	0.974	1.000
FA x CBT-I	cingulum_hippocampus_r	1.48	17.42	0.09	0.932	0.963	1.000
FA x CBT-I	posterior_limb_of_internal_capsule_l	-2.01	24.37	-0.08	0.935	0.963	1.000
FA x CRS	anterior_corona_radiata_l	-2.43	32.10	-0.08	0.940	0.978	1.000
FA x CBT-I	cingulum_cingulate_gyrus_r	1.48	24.30	0.06	0.952	0.963	1.000
FA x CRS	external_capsule_l	-1.43	28.47	-0.05	0.960	0.978	1.000
FA x CBT-I	inferior_cerebellar_peduncle_r	-1.76	38.15	-0.05	0.963	0.963	1.000
FA x CRS	external_capsule_r	0.89	31.99	0.03	0.978	0.978	1.000
FA x combi	fornix_cres_stria_terminalis_can_not_be_resolved_with_current_resolution_l	0.76	33.45	0.02	0.982	0.994	1.000
FA x combi	superior_longitudinal_fasciculus_r	-0.37	34.92	-0.01	0.992	0.994	1.000
FA x combi	pontine_crossing_tract_a_part_of_mcp	0.22	32.21	0.01	0.994	0.994	1.000

Supplementary table 3. Baseline-FA x treatment interaction effects on IDS-SR and ISI at follow-up. Table shows the interaction term statistics extracted from the summary of the full model. Tract names are in FSL format. IDS-SR, inventory depression severity – self report; ISI, insomnia severity index;; FDR, false discovery rate; corr, Bonferoni corrected for correlated measures; FA, fractional anisotropy.

outcome	effect	tract	B	SE	t-value	p	p fdr	p corr
**ids-sr**	MD x CBT-I	inferior_cerebellar_peduncle_r	183.37	76.60	2.39	0.018	0.952	0.740
	MD x combi	anterior_corona_radiata_l	-169.29	77.38	-2.19	0.031	0.952	1.000
	MD x combi	middle_cerebellar_peduncle	-56.93	31.92	-1.78	0.077	0.952	1.000
	MD x CRS	inferior_cerebellar_peduncle_r	113.00	66.67	1.69	0.093	0.952	1.000
	MD x CBT-I	superior_cerebellar_peduncle_r	69.58	43.30	1.61	0.111	0.952	1.000
	MD x combi	cingulum_hippocampus_l	-67.78	43.21	-1.57	0.119	0.952	1.000
	MD x CBT-I	cingulum_hippocampus_r	-63.90	41.49	-1.54	0.126	0.952	1.000
	MD x combi	inferior_cerebellar_peduncle_l	-57.03	38.79	-1.47	0.144	0.952	1.000
	MD x combi	superior_corona_radiata_l	-70.26	47.78	-1.47	0.144	0.952	1.000
	MD x combi	fornix_column_and_body_of_fornix	-48.00	32.88	-1.46	0.147	0.952	1.000
	MD x combi	corticospinal_tract_l	-56.26	38.97	-1.44	0.152	0.952	1.000
	MD x CRS	anterior_corona_radiata_l	-101.74	70.91	-1.43	0.154	0.952	1.000
	MD x CBT-I	medial_lemniscus_l	128.56	92.94	1.38	0.169	0.952	1.000
	MD x combi	posterior_thalamic_radiation_include_op	-47.45	35.75	-1.33	0.187	0.952	1.000
	MD x CBT-I	inferior_cerebellar_peduncle_l	51.58	39.37	1.31	0.193	0.952	1.000
	MD x CBT-I	pontine_crossing_tract_a_part_of_mcp	-47.26	38.20	-1.24	0.219	0.952	1.000
	MD x CBT-I	superior_longitudinal_fasciculus_r	-71.68	57.97	-1.24	0.219	0.952	1.000
	MD x combi	splenium_of_corpus_callosum	-6.47	5.23	-1.24	0.219	0.952	1.000
	MD x combi	corticospinal_tract_r	-35.13	28.47	-1.23	0.220	0.952	1.000
	MD x CRS	anterior_corona_radiata_r	-52.92	43.58	-1.21	0.227	0.952	1.000
	MD x CRS	pontine_crossing_tract_a_part_of_mcp	-43.41	36.19	-1.20	0.233	0.952	1.000
	MD x CBT-I	splenium_of_corpus_callosum	-6.02	5.06	-1.19	0.237	0.952	1.000
	MD x CBT-I	external_capsule_l	-87.77	73.86	-1.19	0.237	0.952	1.000
	MD x combi	body_of_corpus_callosum	-72.12	61.27	-1.18	0.242	0.952	1.000
	MD x CBT-I	anterior_limb_of_internal_capsule_r	66.43	57.03	1.16	0.247	0.952	1.000
	MD x combi	cerebral_peduncle_l	-63.79	57.15	-1.12	0.267	0.952	1.000
	MD x CBT-I	retrolenticular_part_of_internal_capsule	-50.05	46.03	-1.09	0.279	0.952	1.000
	MD x CBT-I	fornix_column_and_body_of_fornix	27.95	25.89	1.08	0.283	0.952	1.000
	MD x CRS	retrolenticular_part_of_internal_capsule	-50.98	47.26	-1.08	0.283	0.952	1.000
	MD x combi	medial_lemniscus_l	-73.43	69.88	-1.05	0.296	0.952	1.000
	MD x CBT-I	superior_cerebellar_peduncle_l	49.74	48.68	1.02	0.309	0.952	1.000
	MD x CBT-I	superior_corona_radiata_r	-61.08	60.06	-1.02	0.311	0.952	1.000
	MD x CBT-I	fornix_cres_stria_terminalis_can_not_be	-65.33	64.42	-1.01	0.313	0.952	1.000
	MD x combi	cingulum_cingulate_gyrus_l	-49.29	48.78	-1.01	0.314	0.952	1.000
	MD x combi	posterior_corona_radiata_l	-42.26	42.30	-1.00	0.320	0.952	1.000
	MD x CBT-I	anterior_corona_radiata_l	-61.73	65.04	-0.95	0.345	0.952	1.000
	MD x CRS	anterior_limb_of_internal_capsule_l	46.34	49.66	0.93	0.353	0.952	1.000
	MD x combi	medial_lemniscus_r	-36.71	39.67	-0.93	0.357	0.952	1.000
	MD x CRS	middle_cerebellar_peduncle	-28.77	31.18	-0.92	0.358	0.952	1.000
	MD x combi	retrolenticular_part_of_internal_capsule	-40.95	44.66	-0.92	0.361	0.952	1.000
	MD x CBT-I	superior_corona_radiata_l	-40.23	44.30	-0.91	0.366	0.952	1.000
	MD x combi	inferior_cerebellar_peduncle_r	61.23	67.98	0.90	0.370	0.952	1.000
	MD x combi	pontine_crossing_tract_a_part_of_mcp	-32.48	36.80	-0.88	0.379	0.952	1.000
	MD x combi	superior_longitudinal_fasciculus_l	-48.13	55.73	-0.86	0.390	0.952	1.000
	MD x combi	uncinate_fasciculus_l	-19.16	22.29	-0.86	0.392	0.952	1.000
	MD x combi	sagittal_stratum_include_inferior_longitu	-54.83	64.37	-0.85	0.396	0.952	1.000
	MD x combi	posterior_limb_of_internal_capsule_l	-45.88	54.83	-0.84	0.404	0.952	1.000

MD x CBT-I	posterior_corona_radiata_l	-33.34	40.41	-0.82	0.411	0.952	1.000
MD x CRS	external_capsule_l	-59.83	72.67	-0.82	0.412	0.952	1.000
MD x combi	fornix_cres_stria_terminalis_can_not_be	-66.86	81.57	-0.82	0.414	0.952	1.000
MD x combi	cerebral_peduncle_r	47.50	58.49	0.81	0.418	0.952	1.000
MD x combi	anterior_corona_radiata_r	-34.32	42.65	-0.80	0.423	0.952	1.000
MD x CRS	fornix_cres_stria_terminalis_can_not_be	-53.21	66.18	-0.80	0.423	0.952	1.000
MD x CBT-I	anterior_corona_radiata_r	-34.38	43.07	-0.80	0.426	0.952	1.000
MD x combi	no_label_skel	-52.87	66.65	-0.79	0.429	0.952	1.000
MD x CRS	superior_corona_radiata_r	-53.08	67.71	-0.78	0.435	0.952	1.000
MD x CRS	superior_cerebellar_peduncle_l	33.56	43.19	0.78	0.439	0.952	1.000
MD x CRS	cerebral_peduncle_r	41.12	53.53	0.77	0.444	0.952	1.000
MD x combi	superior_corona_radiata_r	-44.01	58.65	-0.75	0.455	0.952	1.000
MD x CRS	superior_cerebellar_peduncle_r	28.05	38.20	0.73	0.464	0.952	1.000
MD x CBT-I	uncinate_fasciculus_r	41.93	58.09	0.72	0.472	0.952	1.000
MD x CRS	posterior_thalamic_radiation_include_op	-27.81	38.56	-0.72	0.472	0.952	1.000
MD x CRS	superior_corona_radiata_l	-31.34	45.57	-0.69	0.493	0.952	1.000
MD x CRS	uncinate_fasciculus_l	16.16	23.72	0.68	0.497	0.952	1.000
MD x CBT-I	external_capsule_r	42.70	62.97	0.68	0.499	0.952	1.000
MD x CRS	corticospinal_tract_l	-33.65	50.25	-0.67	0.504	0.952	1.000
MD x CBT-I	posterior_corona_radiata_r	-29.81	44.68	-0.67	0.506	0.952	1.000
MD x CRS	superior_fronto_minus_occipital_fascicu	34.47	51.95	0.66	0.508	0.952	1.000
MD x CRS	medial_lemniscus_r	-28.86	43.55	-0.66	0.509	0.952	1.000
MD x CRS	posterior_thalamic_radiation_include_op	-27.02	40.78	-0.66	0.509	0.952	1.000
MD x combi	posterior_corona_radiata_r	-31.66	48.42	-0.65	0.515	0.952	1.000
MD x combi	retrolenticular_part_of_internal_capsule_	-41.56	64.96	-0.64	0.524	0.952	1.000
MD x CBT-I	no_label_skel	41.27	65.46	0.63	0.530	0.952	1.000
MD x CBT-I	retrolenticular_part_of_internal_capsule_	-37.48	59.62	-0.63	0.531	0.952	1.000
MD x CRS	posterior_corona_radiata_l	-25.64	41.16	-0.62	0.535	0.952	1.000
MD x CBT-I	anterior_limb_of_internal_capsule_l	-38.65	63.08	-0.61	0.541	0.952	1.000
MD x CRS	inferior_cerebellar_peduncle_l	22.75	37.52	0.61	0.546	0.952	1.000
MD x combi	external_capsule_l	-41.29	68.50	-0.60	0.548	0.952	1.000
MD x combi	mean_skel	-47.81	79.49	-0.60	0.549	0.952	1.000
MD x combi	superior_cerebellar_peduncle_l	-31.47	52.47	-0.60	0.550	0.952	1.000
MD x CRS	mean_skel	-50.35	84.93	-0.59	0.554	0.952	1.000
MD x CRS	corticospinal_tract_r	-14.66	24.92	-0.59	0.558	0.952	1.000
MD x CBT-I	cerebral_peduncle_r	34.32	59.07	0.58	0.562	0.952	1.000
MD x CBT-I	posterior_thalamic_radiation_include_op	-22.23	39.25	-0.57	0.572	0.952	1.000
MD x combi	external_capsule_r	35.73	63.40	0.56	0.574	0.952	1.000
MD x CRS	fornix_column_and_body_of_fornix	-13.83	25.07	-0.55	0.582	0.952	1.000
MD x CBT-I	superior_fronto_minus_occipital_fascicu	27.95	51.09	0.55	0.585	0.952	1.000
MD x CBT-I	cingulum_hippocampus_l	-22.73	42.52	-0.53	0.594	0.952	1.000
MD x CRS	medial_lemniscus_l	-39.25	74.24	-0.53	0.598	0.952	1.000
MD x CRS	anterior_limb_of_internal_capsule_r	-35.23	68.89	-0.51	0.610	0.952	1.000
MD x CRS	cingulum_hippocampus_l	-20.66	40.49	-0.51	0.611	0.952	1.000
MD x combi	posterior_thalamic_radiation_include_op	-18.07	35.68	-0.51	0.614	0.952	1.000
MD x CBT-I	corticospinal_tract_l	25.99	51.52	0.50	0.615	0.952	1.000
MD x CRS	splenium_of_corpus_callosum	-2.75	5.67	-0.48	0.629	0.955	1.000
MD x CRS	genu_of_corpus_callosum	23.77	50.73	0.47	0.640	0.955	1.000

MD x CBT-I	mean_skel	-35.86	78.14	-0.46	0.647	0.955	1.000
MD x CRS	body_of_corpus_callosum	-28.89	63.23	-0.46	0.649	0.955	1.000
MD x CRS	cingulum_hippocampus_r	-17.05	38.30	-0.45	0.657	0.955	1.000
MD x CBT-I	cingulum_cingulate_gyrus_l	-21.68	49.35	-0.44	0.661	0.955	1.000
MD x combi	uncinate_fasciculus_r	24.49	58.12	0.42	0.674	0.955	1.000
MD x CRS	uncinate_fasciculus_r	22.85	55.33	0.41	0.680	0.955	1.000
MD x CBT-I	posterior_limb_of_internal_capsule_r	36.33	90.11	0.40	0.688	0.955	1.000
MD x CRS	superior_longitudinal_fasciculus_l	-22.93	57.71	-0.40	0.692	0.955	1.000
MD x CRS	retrolenticular_part_of_internal_capsule_	-23.78	63.95	-0.37	0.711	0.955	1.000
MD x combi	posterior_limb_of_internal_capsule_r	-29.04	78.55	-0.37	0.712	0.955	1.000
MD x combi	anterior_limb_of_internal_capsule_l	-19.45	52.90	-0.37	0.714	0.955	1.000
MD x combi	superior_fronto_minus_occipital_fascicu	-19.66	54.08	-0.36	0.717	0.955	1.000
MD x CRS	superior_longitudinal_fasciculus_r	-26.48	72.95	-0.36	0.717	0.955	1.000
MD x CRS	posterior_limb_of_internal_capsule_r	30.14	84.81	0.36	0.723	0.955	1.000
MD x combi	sagittal_stratum_include_inferior_longitu	12.38	37.05	0.33	0.739	0.961	1.000
MD x CBT-I	body_of_corpus_callosum	-20.83	62.77	-0.33	0.741	0.961	1.000
MD x CBT-I	posterior_limb_of_internal_capsule_l	-15.64	52.78	-0.30	0.767	0.965	1.000
MD x combi	cingulum_cingulate_gyrus_r	-18.31	63.78	-0.29	0.775	0.965	1.000
MD x CBT-I	uncinate_fasciculus_l	-6.46	22.82	-0.28	0.778	0.965	1.000
MD x CBT-I	sagittal_stratum_include_inferior_longitu	-12.70	45.56	-0.28	0.781	0.965	1.000
MD x CBT-I	sagittal_stratum_include_inferior_longitu	-14.70	54.79	-0.27	0.789	0.965	1.000
MD x CRS	cerebral_peduncle_l	-18.99	73.14	-0.26	0.796	0.965	1.000
MD x CRS	sagittal_stratum_include_inferior_longitu	14.48	56.01	0.26	0.797	0.965	1.000
MD x CRS	superior_fronto_minus_occipital_fascicu	-13.72	53.39	-0.26	0.798	0.965	1.000
MD x CRS	fornix_cres_stria_terminalis_can_not_be	12.39	51.59	0.24	0.811	0.965	1.000
MD x CBT-I	medial_lemniscus_r	11.51	48.04	0.24	0.811	0.965	1.000
MD x CBT-I	cingulum_cingulate_gyrus_r	14.57	67.33	0.22	0.829	0.979	1.000
MD x CRS	posterior_corona_radiata_r	-7.70	45.18	-0.17	0.865	0.997	1.000
MD x CBT-I	genu_of_corpus_callosum	-7.58	47.48	-0.16	0.874	0.997	1.000
MD x CBT-I	cerebral_peduncle_l	8.96	58.66	0.15	0.879	0.997	1.000
MD x CRS	posterior_limb_of_internal_capsule_l	7.06	51.62	0.14	0.892	0.997	1.000
MD x combi	genu_of_corpus_callosum	-6.43	47.25	-0.14	0.892	0.997	1.000
MD x CRS	no_label_skel	-6.90	57.86	-0.12	0.905	0.997	1.000
MD x combi	superior_fronto_minus_occipital_fascicu	-6.27	55.52	-0.11	0.910	0.997	1.000
MD x CBT-I	middle_cerebellar_peduncle	3.09	28.91	0.11	0.915	0.997	1.000
MD x CBT-I	superior_longitudinal_fasciculus_l	-5.67	53.77	-0.11	0.916	0.997	1.000
MD x CRS	cingulum_cingulate_gyrus_l	-3.56	47.91	-0.07	0.941	0.997	1.000
MD x CBT-I	corticospinal_tract_r	1.60	26.56	0.06	0.952	0.997	1.000
MD x CRS	sagittal_stratum_include_inferior_longitu	-2.26	40.46	-0.06	0.955	0.997	1.000
MD x combi	fornix_cres_stria_terminalis_can_not_be	-2.55	47.73	-0.05	0.957	0.997	1.000
MD x combi	cingulum_hippocampus_r	-2.09	41.66	-0.05	0.960	0.997	1.000
MD x CBT-I	fornix_cres_stria_terminalis_can_not_be	2.17	47.66	0.05	0.964	0.997	1.000
MD x combi	superior_cerebellar_peduncle_r	-1.68	39.80	-0.04	0.966	0.997	1.000
MD x CBT-I	superior_fronto_minus_occipital_fascicu	-2.57	63.66	-0.04	0.968	0.997	1.000
MD x CRS	external_capsule_r	-2.03	63.35	-0.03	0.975	0.997	1.000
MD x combi	anterior_limb_of_internal_capsule_r	0.88	55.64	0.02	0.987	0.997	1.000
MD x CRS	cingulum_cingulate_gyrus_r	0.75	62.35	0.01	0.990	0.997	1.000
MD x combi	superior_longitudinal_fasciculus_r	0.35	64.72	0.01	0.996	0.997	1.000

	MD x CBT-I	posterior_thalamic_radiation_include_op	-0.11	33.99	0.00	0.997	0.997	1.000
**isi**	MD x combi	inferior_cerebellar_peduncle_l	-54.29	20.39	-2.66	0.009	0.773	0.355
	MD x combi	corticospinal_tract_l	-49.47	21.12	-2.34	0.021	0.773	0.841
	MD x CRS	external_capsule_l	87.05	37.26	2.34	0.021	0.773	0.857
	MD x CRS	posterior_thalamic_radiation_include_op	45.37	20.37	2.23	0.028	0.773	1.000
	MD x CRS	cingulum_cingulate_gyrus_l	55.40	25.10	2.21	0.029	0.773	1.000
	MD x combi	superior_cerebellar_peduncle_r	-41.36	20.89	-1.98	0.050	0.773	1.000
	MD x combi	inferior_cerebellar_peduncle_r	-73.40	37.85	-1.94	0.055	0.773	1.000
	MD x CRS	posterior_limb_of_internal_capsule_r	85.54	44.69	1.91	0.058	0.773	1.000
	MD x CBT-I	posterior_limb_of_internal_capsule_r	89.29	47.51	1.88	0.063	0.773	1.000
	MD x CRS	retrolenticular_part_of_internal_capsule	59.89	32.92	1.82	0.072	0.773	1.000
	MD x combi	middle_cerebellar_peduncle	-30.62	17.27	-1.77	0.079	0.773	1.000
	MD x CRS	genu_of_corpus_callosum	44.82	26.30	1.70	0.091	0.773	1.000
	MD x CRS	mean_skel	74.86	45.42	1.65	0.102	0.773	1.000
	MD x CBT-I	cingulum_hippocampus_r	37.36	22.77	1.64	0.104	0.773	1.000
	MD x CRS	cingulum_hippocampus_r	34.17	20.97	1.63	0.106	0.773	1.000
	MD x CRS	posterior_limb_of_internal_capsule_l	43.36	26.76	1.62	0.108	0.773	1.000
	MD x CRS	anterior_corona_radiata_l	61.50	38.06	1.62	0.109	0.773	1.000
	MD x CRS	superior_corona_radiata_r	57.48	35.82	1.60	0.111	0.773	1.000
	MD x combi	medial_lemniscus_l	-60.24	37.86	-1.59	0.114	0.773	1.000
	MD x CBT-I	fornix_column_and_body_of_fornix	21.86	13.87	1.58	0.118	0.773	1.000
	MD x combi	medial_lemniscus_r	-33.64	21.46	-1.57	0.120	0.773	1.000
	MD x CRS	sagittal_stratum_include_inferior_longitu	33.33	21.57	1.55	0.125	0.773	1.000
	MD x CRS	cingulum_cingulate_gyrus_r	49.19	32.37	1.52	0.131	0.773	1.000
	MD x CRS	posterior_thalamic_radiation_include_op	32.56	21.56	1.51	0.134	0.773	1.000
	MD x CBT-I	superior_fronto_minus_occipital_fascicu	50.03	33.73	1.48	0.141	0.773	1.000
	MD x CBT-I	medial_lemniscus_l	-74.47	50.50	-1.47	0.143	0.773	1.000
	MD x CRS	fornix_cres_stria_terminalis_can_not_be	38.78	27.07	1.43	0.155	0.773	1.000
	MD x CRS	anterior_limb_of_internal_capsule_r	52.56	36.78	1.43	0.156	0.773	1.000
	MD x combi	splenium_of_corpus_callosum	-3.89	2.74	-1.42	0.159	0.773	1.000
	MD x CBT-I	fornix_cres_stria_terminalis_can_not_be	35.52	25.18	1.41	0.161	0.773	1.000
	MD x CRS	retrolenticular_part_of_internal_capsule	32.65	24.17	1.35	0.179	0.803	1.000
	MD x CRS	cingulum_hippocampus_l	28.49	21.40	1.33	0.186	0.803	1.000
	MD x CRS	body_of_corpus_callosum	44.31	33.39	1.33	0.187	0.803	1.000
	MD x CBT-I	external_capsule_r	44.64	33.82	1.32	0.189	0.803	1.000
	MD x CRS	cerebral_peduncle_l	49.70	38.99	1.27	0.205	0.837	1.000
	MD x CRS	anterior_limb_of_internal_capsule_l	33.60	27.68	1.21	0.227	0.837	1.000
	MD x CRS	superior_corona_radiata_l	28.92	23.84	1.21	0.228	0.837	1.000
	MD x CBT-I	genu_of_corpus_callosum	29.28	24.61	1.19	0.237	0.837	1.000
	MD x CRS	uncinate_fasciculus_r	35.37	29.79	1.19	0.238	0.837	1.000
	MD x combi	sagittal_stratum_include_inferior_longitu	23.24	19.60	1.19	0.238	0.837	1.000
	MD x CBT-I	retrolenticular_part_of_internal_capsule	28.07	23.75	1.18	0.240	0.837	1.000
	MD x combi	pontine_crossing_tract_a_part_of_mcp	-20.85	18.45	-1.13	0.261	0.837	1.000
	MD x CRS	inferior_cerebellar_peduncle_l	-21.69	19.50	-1.11	0.268	0.837	1.000
	MD x CBT-I	inferior_cerebellar_peduncle_l	-22.74	20.52	-1.11	0.270	0.837	1.000
	MD x CBT-I	splenium_of_corpus_callosum	-2.90	2.63	-1.10	0.273	0.837	1.000
	MD x combi	posterior_limb_of_internal_capsule_r	44.50	41.63	1.07	0.287	0.837	1.000
	MD x CBT-I	superior_cerebellar_peduncle_r	-23.34	22.39	-1.04	0.299	0.837	1.000

MD x combi	superior_corona_radiata_r	32.30	31.02	1.04	0.300	0.837	1.000
MD x CRS	superior_cerebellar_peduncle_r	-20.00	19.56	-1.02	0.309	0.837	1.000
MD x CBT-I	uncinate_fasciculus_l	-12.24	12.07	-1.01	0.313	0.837	1.000
MD x combi	superior_cerebellar_peduncle_l	-28.90	28.63	-1.01	0.315	0.837	1.000
MD x CBT-I	cingulum_cingulate_gyrus_r	35.19	35.27	1.00	0.321	0.837	1.000
MD x CRS	external_capsule_r	34.23	34.46	0.99	0.323	0.837	1.000
MD x CBT-I	mean_skel	41.80	42.08	0.99	0.323	0.837	1.000
MD x CBT-I	cingulum_cingulate_gyrus_l	25.41	25.78	0.99	0.326	0.837	1.000
MD x combi	cerebral_peduncle_r	-31.20	31.70	-0.98	0.327	0.837	1.000
MD x CRS	superior_longitudinal_fasciculus_r	36.84	39.09	0.94	0.348	0.837	1.000
MD x CBT-I	superior_cerebellar_peduncle_l	-24.43	26.57	-0.92	0.360	0.837	1.000
MD x CRS	superior_fronto_minus_occipital_fascicu	26.22	28.53	0.92	0.360	0.837	1.000
MD x combi	external_capsule_r	30.37	34.27	0.89	0.377	0.837	1.000
MD x combi	cingulum_hippocampus_l	-20.33	22.96	-0.89	0.378	0.837	1.000
MD x CRS	posterior_corona_radiata_r	20.52	23.89	0.86	0.392	0.837	1.000
MD x CRS	fornix_column_and_body_of_fornix	11.46	13.41	0.85	0.394	0.837	1.000
MD x CBT-I	uncinate_fasciculus_r	26.37	31.08	0.85	0.398	0.837	1.000
MD x CBT-I	superior_fronto_minus_occipital_fascicu	-22.51	27.27	-0.83	0.411	0.837	1.000
MD x combi	posterior_thalamic_radiation_include_op	15.50	18.92	0.82	0.414	0.837	1.000
MD x combi	cerebral_peduncle_l	-24.43	30.42	-0.80	0.424	0.837	1.000
MD x CRS	superior_fronto_minus_occipital_fascicu	22.27	27.83	0.80	0.425	0.837	1.000
MD x CBT-I	medial_lemniscus_r	-20.49	25.66	-0.80	0.426	0.837	1.000
MD x CBT-I	corticospinal_tract_l	-22.30	27.95	-0.80	0.427	0.837	1.000
MD x CRS	pontine_crossing_tract_a_part_of_mcp	14.55	18.38	0.79	0.430	0.837	1.000
MD x CBT-I	inferior_cerebellar_peduncle_r	-32.60	42.47	-0.77	0.444	0.837	1.000
MD x CBT-I	corticospinal_tract_r	10.65	14.24	0.75	0.456	0.837	1.000
MD x combi	superior_fronto_minus_occipital_fascicu	-22.05	29.84	-0.74	0.462	0.837	1.000
MD x CBT-I	anterior_corona_radiata_r	16.46	22.67	0.73	0.469	0.837	1.000
MD x combi	retrolenticular_part_of_internal_capsule	16.41	22.86	0.72	0.474	0.837	1.000
MD x CBT-I	external_capsule_l	27.58	38.60	0.71	0.476	0.837	1.000
MD x combi	cingulum_cingulate_gyrus_r	-24.05	33.68	-0.71	0.477	0.837	1.000
MD x CRS	posterior_corona_radiata_l	15.75	22.21	0.71	0.480	0.837	1.000
MD x CBT-I	retrolenticular_part_of_internal_capsule	20.87	30.49	0.68	0.495	0.837	1.000
MD x CBT-I	anterior_limb_of_internal_capsule_r	20.42	30.34	0.67	0.502	0.837	1.000
MD x combi	anterior_corona_radiata_r	14.81	22.43	0.66	0.511	0.837	1.000
MD x CBT-I	body_of_corpus_callosum	-21.58	32.99	-0.65	0.514	0.837	1.000
MD x combi	superior_fronto_minus_occipital_fascicu	18.83	28.83	0.65	0.515	0.837	1.000
MD x combi	uncinate_fasciculus_l	-7.70	11.81	-0.65	0.516	0.837	1.000
MD x CBT-I	pontine_crossing_tract_a_part_of_mcp	12.34	19.17	0.64	0.521	0.837	1.000
MD x combi	no_label_skel	-23.24	36.14	-0.64	0.521	0.837	1.000
MD x CRS	fornix_cres_stria_terminalis_can_not_be	22.95	35.99	0.64	0.525	0.837	1.000
MD x CBT-I	sagittal_stratum_include_inferior_longitu	15.51	24.46	0.63	0.527	0.837	1.000
MD x CRS	medial_lemniscus_r	-14.55	23.13	-0.63	0.531	0.837	1.000
MD x combi	fornix_column_and_body_of_fornix	-11.09	17.75	-0.62	0.533	0.837	1.000
MD x combi	cingulum_hippocampus_r	14.05	22.57	0.62	0.535	0.837	1.000
MD x CRS	middle_cerebellar_peduncle	-10.06	16.66	-0.60	0.547	0.838	1.000
MD x combi	genu_of_corpus_callosum	14.79	24.48	0.60	0.547	0.838	1.000
MD x CRS	uncinate_fasciculus_l	7.45	12.63	0.59	0.557	0.844	1.000

MD x combi	superior_corona_radiata_l	-14.38	25.14	-0.57	0.569	0.853	1.000
MD x CRS	anterior_corona_radiata_r	12.44	22.85	0.54	0.587	0.872	1.000
MD x combi	posterior_limb_of_internal_capsule_l	14.69	28.71	0.51	0.610	0.888	1.000
MD x combi	posterior_corona_radiata_l	11.33	22.93	0.49	0.622	0.888	1.000
MD x combi	uncinate_fasciculus_r	-14.94	31.04	-0.48	0.631	0.888	1.000
MD x CRS	splenium_of_corpus_callosum	1.44	3.02	0.48	0.633	0.888	1.000
MD x combi	mean_skel	20.51	42.88	0.48	0.633	0.888	1.000
MD x CBT-I	superior_longitudinal_fasciculus_r	14.66	31.07	0.47	0.638	0.888	1.000
MD x combi	corticospinal_tract_r	-7.15	15.32	-0.47	0.641	0.888	1.000
MD x CRS	corticospinal_tract_r	5.65	13.37	0.42	0.673	0.898	1.000
MD x combi	posterior_corona_radiata_r	-10.73	25.60	-0.42	0.676	0.898	1.000
MD x CBT-I	posterior_thalamic_radiation_include_op	7.49	18.04	0.42	0.679	0.898	1.000
MD x combi	anterior_corona_radiata_l	16.56	41.36	0.40	0.690	0.898	1.000
MD x CBT-I	superior_longitudinal_fasciculus_l	-11.52	28.86	-0.40	0.691	0.898	1.000
MD x CBT-I	posterior_thalamic_radiation_include_op	-8.35	20.98	-0.40	0.692	0.898	1.000
MD x combi	cingulum_cingulate_gyrus_l	-10.11	25.48	-0.40	0.692	0.898	1.000
MD x combi	body_of_corpus_callosum	-12.48	32.27	-0.39	0.700	0.898	1.000
MD x CBT-I	cerebral_peduncle_r	12.13	32.10	0.38	0.706	0.898	1.000
MD x CBT-I	superior_corona_radiata_l	-8.63	23.23	-0.37	0.711	0.898	1.000
MD x CBT-I	superior_corona_radiata_r	10.35	31.64	0.33	0.744	0.920	1.000
MD x combi	sagittal_stratum_include_inferior_longitu	-11.43	35.43	-0.32	0.748	0.920	1.000
MD x combi	anterior_limb_of_internal_capsule_l	9.29	29.38	0.32	0.752	0.920	1.000
MD x combi	fornix_cres_stria_terminalis_can_not_be	-7.74	25.06	-0.31	0.758	0.920	1.000
MD x combi	fornix_cres_stria_terminalis_can_not_be	13.34	44.67	0.30	0.766	0.920	1.000
MD x CRS	medial_lemniscus_l	-11.46	38.48	-0.30	0.766	0.920	1.000
MD x CRS	no_label_skel	8.91	31.27	0.29	0.776	0.923	1.000
MD x CBT-I	posterior_limb_of_internal_capsule_l	-7.46	27.60	-0.27	0.787	0.923	1.000
MD x combi	external_capsule_l	-9.66	35.83	-0.27	0.788	0.923	1.000
MD x CBT-I	anterior_limb_of_internal_capsule_l	8.85	34.96	0.25	0.801	0.929	1.000
MD x combi	posterior_thalamic_radiation_include_op	4.66	18.98	0.25	0.806	0.929	1.000
MD x combi	anterior_limb_of_internal_capsule_r	-6.15	29.66	-0.21	0.836	0.956	1.000
MD x CRS	sagittal_stratum_include_inferior_longitu	5.57	30.88	0.18	0.857	0.969	1.000
MD x CBT-I	sagittal_stratum_include_inferior_longitu	-5.25	30.42	-0.17	0.863	0.969	1.000
MD x CRS	superior_longitudinal_fasciculus_l	-4.96	30.93	-0.16	0.873	0.969	1.000
MD x combi	superior_longitudinal_fasciculus_r	-4.43	34.55	-0.13	0.898	0.969	1.000
MD x CRS	superior_cerebellar_peduncle_l	3.00	23.62	0.13	0.899	0.969	1.000
MD x CBT-I	middle_cerebellar_peduncle	-1.76	15.37	-0.11	0.909	0.969	1.000
MD x CBT-I	cingulum_hippocampus_l	-2.54	22.31	-0.11	0.910	0.969	1.000
MD x CBT-I	no_label_skel	-3.26	35.39	-0.09	0.927	0.969	1.000
MD x combi	superior_longitudinal_fasciculus_l	2.72	30.15	0.09	0.928	0.969	1.000
MD x CRS	inferior_cerebellar_peduncle_r	2.91	36.04	0.08	0.936	0.969	1.000
MD x CRS	corticospinal_tract_l	2.17	27.06	0.08	0.936	0.969	1.000
MD x CBT-I	anterior_corona_radiata_l	2.62	34.89	0.08	0.940	0.969	1.000
MD x CBT-I	fornix_cres_stria_terminalis_can_not_be	2.52	35.08	0.07	0.943	0.969	1.000
MD x CBT-I	cerebral_peduncle_l	-1.76	31.28	-0.06	0.955	0.969	1.000
MD x combi	retrolenticular_part_of_internal_capsule	-1.67	33.07	-0.05	0.960	0.969	1.000
MD x CBT-I	posterior_corona_radiata_r	1.18	23.61	0.05	0.960	0.969	1.000
MD x CRS	cerebral_peduncle_r	-1.37	29.10	-0.05	0.963	0.969	1.000

MD x CBT-I	posterior_corona_radiata_l	-0.52	21.89	-0.02	0.981	0.981	1.000

Supplementary table 4. Baseline-MD x treatment interaction effects on IDS-SR and ISI at follow-up. Table shows the interaction term statistics extracted from the summary of the full model. Tract names are in FSL format. IDS-SR, inventory depression severity – self report; ISI, insomnia severity index;; FDR, false discovery rate; corr, Bonferoni corrected for correlated measures; MD, mead diffusivity.

outcome	effect	tract	B	SE	t-value	p	p fdr	p corr
**ids-sr**	FA x intervention	retrolenticular_part_of_internal_capsule_l	149.04	44.55	3.35	0.001	0.053	0.045
	FA x intervention	anterior_corona_radiata_r	116.73	43.84	2.66	0.009	0.213	0.358
	FA x intervention	splenium_of_corpus_callosum	32.78	13.69	2.39	0.018	0.266	0.738
	FA x intervention	cerebral_peduncle_l	112.48	48.51	2.32	0.022	0.266	0.895
	FA x intervention	fornix_cres_stria_terminalis_can_not_be_resolved_with_current_resolution_l	101.88	47.71	2.14	0.035	0.326	1.000
	FA x intervention	retrolenticular_part_of_internal_capsule_r	79.91	38.61	2.07	0.041	0.326	1.000
	FA x intervention	anterior_limb_of_internal_capsule_r	86.49	47.68	1.81	0.072	0.436	1.000
	FA x intervention	mean_skel	129.47	76.46	1.69	0.093	0.436	1.000
	FA x intervention	inferior_cerebellar_peduncle_r	-114.65	68.36	-1.68	0.096	0.436	1.000
	FA x intervention	posterior_limb_of_internal_capsule_l	63.81	38.37	1.66	0.099	0.436	1.000
	FA x intervention	superior_longitudinal_fasciculus_r	85.06	51.38	1.66	0.101	0.436	1.000
	FA x intervention	medial_lemniscus_l	-98.24	61.01	-1.61	0.110	0.436	1.000
	FA x intervention	superior_corona_radiata_r	66.61	42.31	1.57	0.118	0.436	1.000
	FA x intervention	cerebral_peduncle_r	70.90	48.33	1.47	0.145	0.463	1.000
	FA x intervention	posterior_thalamic_radiation_include_optic_radiation_l	52.33	35.99	1.45	0.149	0.463	1.000
	FA x intervention	pontine_crossing_tract_a_part_of_mcp	76.93	53.67	1.43	0.154	0.463	1.000
	FA x intervention	cingulum_cingulate_gyrus_r	57.88	41.65	1.39	0.167	0.472	1.000
	FA x intervention	inferior_cerebellar_peduncle_l	55.37	46.67	1.19	0.238	0.603	1.000
	FA x intervention	posterior_thalamic_radiation_include_optic_radiation_r	45.66	38.54	1.18	0.239	0.603	1.000
	FA x intervention	posterior_limb_of_internal_capsule_r	59.39	51.50	1.15	0.251	0.603	1.000
	FA x intervention	middle_cerebellar_peduncle	35.22	32.78	1.07	0.285	0.620	1.000
	FA x intervention	sagittal_stratum_include_inferior_longitidinal_fasciculus_and_inferior_fronto_minus_occipital_fasciculus_r	41.80	39.83	1.05	0.296	0.620	1.000
	FA x intervention	body_of_corpus_callosum	56.37	53.80	1.05	0.297	0.620	1.000
	FA x intervention	no_label_skel	56.31	60.32	0.93	0.353	0.685	1.000
	FA x intervention	anterior_corona_radiata_l	43.12	46.99	0.92	0.361	0.685	1.000
	FA x intervention	superior_longitudinal_fasciculus_l	38.86	43.96	0.88	0.379	0.685	1.000
	FA x intervention	superior_cerebellar_peduncle_l	45.27	53.41	0.85	0.398	0.685	1.000
	FA x intervention	posterior_corona_radiata_l	36.11	42.72	0.85	0.400	0.685	1.000
	FA x intervention	fornix_cres_stria_terminalis_can_not_be_resolved_with_current_resolution_r	29.49	37.69	0.78	0.436	0.719	1.000
	FA x intervention	superior_cerebellar_peduncle_r	37.89	49.89	0.76	0.449	0.719	1.000
	FA x intervention	cingulum_hippocampus_r	19.28	27.36	0.70	0.482	0.724	1.000
	FA x intervention	uncinate_fasciculus_l	-20.91	31.15	-0.67	0.503	0.724	1.000
	FA x intervention	external_capsule_l	28.95	43.89	0.66	0.511	0.724	1.000
	FA x intervention	superior_fronto_minus_occipital_fasciculus_could_be_a_part_of_anterior_internal_capsule_r	23.48	35.73	0.66	0.512	0.724	1.000
	FA x intervention	corticospinal_tract_r	22.67	36.35	0.62	0.534	0.733	1.000
	FA x intervention	fornix_column_and_body_of_fornix	24.48	43.32	0.57	0.573	0.740	1.000
	FA x intervention	genu_of_corpus_callosum	27.93	52.48	0.53	0.596	0.740	1.000
	FA x intervention	uncinate_fasciculus_r	-11.51	21.65	-0.53	0.596	0.740	1.000
	FA x intervention	sagittal_stratum_include_inferior_longitidinal_fasciculus_and_inferior_fronto_minus_occipital_fasciculus_l	25.96	49.49	0.52	0.601	0.740	1.000
	FA x intervention	corticospinal_tract_l	-30.03	61.88	-0.49	0.628	0.741	1.000
	FA x intervention	cingulum_cingulate_gyrus_l	-16.39	34.26	-0.48	0.633	0.741	1.000

	FA x intervention	cingulum_hippocampus_l	17.77	42.40	0.42	0.676	0.772	1.000
	FA x intervention	superior_corona_radiata_l	-17.66	48.46	-0.36	0.716	0.799	1.000
	FA x intervention	medial_lemniscus_r	18.28	56.07	0.33	0.745	0.813	1.000
	FA x intervention	external_capsule_r	-13.22	48.40	-0.27	0.785	0.824	1.000
	FA x intervention	superior_fronto_minus_occipital_fasciculus_could_be_a_part_of_anterior_internal_capsule_l	-7.13	28.50	-0.25	0.803	0.824	1.000
	FA x intervention	posterior_corona_radiata_r	11.16	45.53	0.25	0.807	0.824	1.000
	FA x intervention	anterior_limb_of_internal_capsule_l	-9.47	53.78	-0.18	0.861	0.861	1.000
**isi**	FA x intervention	inferior_cerebellar_peduncle_l	57.16	23.63	2.42	0.017	0.420	0.690
	FA x intervention	superior_cerebellar_peduncle_r	56.77	24.45	2.32	0.022	0.420	0.887
	FA x intervention	posterior_limb_of_internal_capsule_r	-61.90	27.50	-2.25	0.026	0.420	1.000
	FA x intervention	anterior_limb_of_internal_capsule_l	-52.37	29.04	-1.80	0.074	0.719	1.000
	FA x intervention	uncinate_fasciculus_l	-30.00	16.69	-1.80	0.075	0.719	1.000
	FA x intervention	middle_cerebellar_peduncle	24.90	15.56	1.60	0.112	0.849	1.000
	FA x intervention	posterior_corona_radiata_l	-34.85	23.25	-1.50	0.137	0.849	1.000
	FA x intervention	superior_corona_radiata_l	-38.89	26.46	-1.47	0.144	0.849	1.000
	FA x intervention	fornix_cres_stria_terminalis_can_not_be_resolved_with_current_resolution_r	-26.96	20.48	-1.32	0.191	0.849	1.000
	FA x intervention	sagittal_stratum_include_inferior_longitidinal_fasciculus_and_inferior_fronto_minus_occipital_fasciculus_r	-28.30	21.53	-1.31	0.191	0.849	1.000
	FA x intervention	anterior_corona_radiata_l	-33.12	25.38	-1.31	0.194	0.849	1.000
	FA x intervention	anterior_corona_radiata_r	-28.23	23.64	-1.19	0.235	0.878	1.000
	FA x intervention	external_capsule_r	-30.76	26.57	-1.16	0.249	0.878	1.000
	FA x intervention	mean_skel	-43.12	41.45	-1.04	0.300	0.878	1.000
	FA x intervention	medial_lemniscus_l	-33.00	32.99	-1.00	0.319	0.878	1.000
	FA x intervention	posterior_thalamic_radiation_include_optic_radiation_l	-19.29	19.91	-0.97	0.335	0.878	1.000
	FA x intervention	inferior_cerebellar_peduncle_r	-33.49	37.17	-0.90	0.369	0.878	1.000
	FA x intervention	posterior_thalamic_radiation_include_optic_radiation_r	-18.35	20.75	-0.88	0.379	0.878	1.000
	FA x intervention	anterior_limb_of_internal_capsule_r	-23.35	26.53	-0.88	0.381	0.878	1.000
	FA x intervention	fornix_cres_stria_terminalis_can_not_be_resolved_with_current_resolution_l	22.26	27.20	0.82	0.415	0.878	1.000
	FA x intervention	retrolenticular_part_of_internal_capsule_r	-17.06	21.56	-0.79	0.430	0.878	1.000
	FA x intervention	superior_longitudinal_fasciculus_l	-18.39	23.60	-0.78	0.437	0.878	1.000
	FA x intervention	pontine_crossing_tract_a_part_of_mcp	-21.52	27.85	-0.77	0.441	0.878	1.000
	FA x intervention	splenium_of_corpus_callosum	4.90	7.55	0.65	0.518	0.878	1.000
	FA x intervention	body_of_corpus_callosum	18.32	29.77	0.62	0.540	0.878	1.000
	FA x intervention	cingulum_cingulate_gyrus_l	-11.28	19.14	-0.59	0.557	0.878	1.000
	FA x intervention	superior_fronto_minus_occipital_fasciculus_could_be_a_part_of_anterior_internal_capsule_r	-10.91	19.48	-0.56	0.577	0.878	1.000
	FA x intervention	cerebral_peduncle_r	-14.87	27.23	-0.55	0.586	0.878	1.000
	FA x intervention	retrolenticular_part_of_internal_capsule_l	-12.17	24.68	-0.49	0.623	0.878	1.000
	FA x intervention	superior_fronto_minus_occipital_fasciculus_could_be_a_part_of_anterior_internal_capsule_l	7.84	15.91	0.49	0.623	0.878	1.000
	FA x intervention	genu_of_corpus_callosum	-13.79	28.21	-0.49	0.626	0.878	1.000
	FA x intervention	posterior_corona_radiata_r	-12.22	24.99	-0.49	0.626	0.878	1.000

FA x intervention	sagittal_stratum_include_inferior_longitidinal_fasciculus_and_inferior_fronto_minus_occipital_fasciculus_l	-13.04	27.20	-0.48	0.633	0.878	1.000
FA x intervention	superior_longitudinal_fasciculus_r	12.60	28.48	0.44	0.659	0.878	1.000
FA x intervention	uncinate_fasciculus_r	-5.18	11.92	-0.43	0.665	0.878	1.000
FA x intervention	external_capsule_l	-9.50	23.95	-0.40	0.692	0.878	1.000
FA x intervention	corticospinal_tract_l	-13.31	34.03	-0.39	0.696	0.878	1.000
FA x intervention	superior_corona_radiata_r	-8.87	23.65	-0.37	0.708	0.878	1.000
FA x intervention	cingulum_hippocampus_r	-5.20	15.10	-0.34	0.731	0.878	1.000
FA x intervention	fornix_column_and_body_of_fornix	6.88	20.02	0.34	0.732	0.878	1.000
FA x intervention	posterior_limb_of_internal_capsule_l	-6.03	21.45	-0.28	0.779	0.879	1.000
FA x intervention	cerebral_peduncle_l	-7.53	27.19	-0.28	0.782	0.879	1.000
FA x intervention	medial_lemniscus_r	-7.99	30.78	-0.26	0.796	0.879	1.000
FA x intervention	cingulum_cingulate_gyrus_r	5.54	22.49	0.25	0.806	0.879	1.000
FA x intervention	corticospinal_tract_r	-2.58	17.97	-0.14	0.886	0.930	1.000
FA x intervention	cingulum_hippocampus_l	-2.93	23.46	-0.12	0.901	0.930	1.000
FA x intervention	superior_cerebellar_peduncle_l	-3.33	29.56	-0.11	0.910	0.930	1.000
FA x intervention	no_label_skel	0.95	32.94	0.03	0.977	0.977	1.000

Supplementary table 5. Baseline-FA x 'any intervention' interaction effects on IDS-SR and ISI at follow-up. Table shows the interaction term statistics extracted from the summary of the full model. Tract names are in FSL format. IDS-SR, inventory depression severity – self report; ISI, insomnia severity index; FDR, false discovery rate; corr, Bonferoni corrected for correlated measures; FA, fractional anisotropy.

tract	n	CRS					CBT-I				
		β	SE	p	p fdr	p corr	β	SE	p	p fdr	p corr
mean_skel	115	-0.15	0.11	0.188	0.948	1.000	-0.27	0.12	0.023	0.829	0.803
no_label_skel	116	-0.41	0.2	0.045	0.948	1.000	-0.18	0.21	0.392	0.829	1.000
posterior_thalamic_radiation_include_optic_radiation_l	117	-0.15	0.13	0.249	0.948	1.000	-0.27	0.14	0.05	0.829	1.000
middle_cerebellar_peduncle	117	-0.4	0.21	0.057	0.948	1.000	-0.41	0.21	0.056	0.829	1.000
superior_corona_radiata_r	116	0.04	0.1	0.692	0.948	1.000	0.11	0.1	0.292	0.829	1.000
splenium_of_corpus_callosum	116	-0.07	0.11	0.493	0.948	1.000	-0.04	0.11	0.709	0.896	1.000
medial_lemniscus_r	117	-0.28	0.19	0.14	0.948	1.000	-0.17	0.2	0.393	0.829	1.000
posterior_corona_radiata_l	117	0.03	0.11	0.789	0.948	1.000	-0.17	0.11	0.122	0.829	1.000
medial_lemniscus_l	116	-0.14	0.21	0.51	0.948	1.000	-0.33	0.22	0.133	0.829	1.000
superior_fronto_minus_occipital_fasciculus_could_be_a_part_of_anterior_internal_capsule_l	117	-0.08	0.12	0.533	0.948	1.000	-0.19	0.13	0.134	0.829	1.000
posterior_limb_of_internal_capsule_l	117	-0.11	0.14	0.42	0.948	1.000	-0.1	0.15	0.502	0.829	1.000
anterior_limb_of_internal_capsule_r	117	0.02	0.19	0.923	0.948	1.000	0.28	0.19	0.143	0.829	1.000
fornix_column_and_body_of_fornix	115	0.03	0.24	0.898	0.948	1.000	0.38	0.26	0.145	0.829	1.000
superior_longitudinal_fasciculus_r	117	0.08	0.1	0.464	0.948	1.000	0.09	0.11	0.431	0.829	1.000
retrolenticular_part_of_internal_capsule_r	116	-0.09	0.17	0.598	0.948	1.000	-0.21	0.17	0.223	0.829	1.000
corticospinal_tract_l	115	-0.13	0.2	0.531	0.948	1.000	-0.14	0.21	0.506	0.829	1.000
anterior_corona_radiata_l	116	0.13	0.11	0.235	0.948	1.000	0.08	0.11	0.451	0.829	1.000
inferior_cerebellar_peduncle_r	116	-0.08	0.24	0.74	0.948	1.000	-0.17	0.25	0.501	0.829	1.000
superior_fronto_minus_occipital_fasciculus_could_be_a_part_of_anterior_internal_capsule_r	117	-0.07	0.17	0.698	0.948	1.000	0.04	0.18	0.817	0.896	1.000
posterior_corona_radiata_r	117	-0.08	0.12	0.502	0.948	1.000	-0.08	0.13	0.508	0.829	1.000
external_capsule_r	117	0.12	0.14	0.372	0.948	1.000	0.13	0.14	0.365	0.829	1.000
body_of_corpus_callosum	117	0.01	0.19	0.948	0.948	1.000	-0.18	0.19	0.354	0.829	1.000
external_capsule_l	117	-0.06	0.11	0.56	0.948	1.000	-0.1	0.11	0.37	0.829	1.000
superior_corona_radiata_l	117	0.1	0.11	0.389	0.948	1.000	-0.1	0.12	0.379	0.829	1.000
fornix_cres_stria_terminalis_can_not_be_resolved_with_current_resolution_l	117	0.05	0.11	0.664	0.948	1.000	-0.09	0.11	0.406	0.829	1.000
sagittal_stratum_include_inferior_longitidinal_fasciculus_and_inferior_fronto_minus_occipital_fasciculus_r	117	0.02	0.15	0.913	0.948	1.000	-0.13	0.16	0.417	0.829	1.000
uncinate_fasciculus_r	117	0.13	0.17	0.439	0.948	1.000	0.14	0.18	0.449	0.829	1.000
retrolenticular_part_of_internal_capsule_l	117	-0.01	0.09	0.929	0.948	1.000	-0.07	0.1	0.44	0.829	1.000
posterior_thalamic_radiation_include_optic_radiation_r	117	0.05	0.11	0.687	0.948	1.000	-0.09	0.12	0.447	0.829	1.000
pontine_crossing_tract_a_part_of_mcp	117	0.07	0.14	0.612	0.948	1.000	-0.04	0.15	0.81	0.896	1.000
cingulum_hippocampus_l	117	-0.08	0.17	0.619	0.948	1.000	-0.12	0.17	0.485	0.829	1.000
superior_longitudinal_fasciculus_l	117	-0.1	0.14	0.48	0.948	1.000	-0.09	0.15	0.565	0.834	1.000
uncinate_fasciculus_l	116	0.03	0.09	0.768	0.948	1.000	-0.06	0.09	0.495	0.829	1.000
genu_of_corpus_callosum	115	0.03	0.13	0.826	0.948	1.000	-0.03	0.13	0.801	0.896	1.000
posterior_limb_of_internal_capsule_r	117	0.12	0.19	0.507	0.948	1.000	0.03	0.19	0.895	0.896	1.000
sagittal_stratum_include_inferior_longitidinal_fasciculus_and_inferior_fronto_minus_occipital_fasciculus_l	117	-0.08	0.12	0.528	0.948	1.000	-0.08	0.13	0.535	0.829	1.000
cingulum_cingulate_gyrus_l	117	-0.01	0.18	0.934	0.948	1.000	-0.12	0.18	0.528	0.829	1.000
cerebral_peduncle_l	116	0.02	0.16	0.895	0.948	1.000	0.06	0.16	0.715	0.896	1.000
anterior_limb_of_internal_capsule_l	117	0.02	0.19	0.9	0.948	1.000	0.04	0.2	0.835	0.896	1.000
anterior_corona_radiata_r	116	0.04	0.1	0.687	0.948	1.000	-0.04	0.1	0.688	0.896	1.000
cingulum_cingulate_gyrus_r	117	0.03	0.12	0.803	0.948	1.000	0.07	0.12	0.573	0.834	1.000
corticospinal_tract_r	116	-0.13	0.24	0.603	0.948	1.000	0.05	0.26	0.847	0.896	1.000
inferior_cerebellar_peduncle_l	117	0.07	0.17	0.689	0.948	1.000	-0.09	0.18	0.619	0.873	1.000
cerebral_peduncle_r	116	0.1	0.24	0.69	0.948	1.000	0.05	0.25	0.847	0.896	1.000
fornix_cres_stria_terminalis_can_not_be_resolved_with_current_resolution_r	117	0.05	0.18	0.795	0.948	1.000	-0.08	0.18	0.662	0.896	1.000
cingulum_hippocampus_r	117	-0.08	0.21	0.685	0.948	1.000	0.04	0.22	0.858	0.896	1.000

superior_cerebellar_peduncle_l	117	-0.07	0.23	0.756	0.948	1.000	-0.08	0.24	0.744	0.896	1.000
superior_cerebellar_peduncle_r	117	0.05	0.19	0.777	0.948	1.000	-0.03	0.19	0.896	0.896	1.000

Supplementary table 6. The effect of insomnia interventions on tract FA. Standerdized effect size (β) for each insomnia intervention compared to the no treatment group at week 7 (T1). FA, fractional anisotropy.

		CBT-I+CRS				
**tract**	**n**	**β**	**SE**	**p**	**p fdr**	**p corr**
mean_skel	115	-0.17	0.12	0.15	0.969	1.000
no_label_skel	116	-0.31	0.21	0.138	0.969	1.000
posterior_thalamic_radiation_include_optic_radiation_l	117	-0.17	0.13	0.214	0.969	1.000
middle_cerebellar_peduncle	117	-0.29	0.21	0.176	0.969	1.000
superior_corona_radiata_r	116	0.18	0.1	0.073	0.969	1.000
splenium_of_corpus_callosum	116	-0.19	0.11	0.092	0.969	1.000
medial_lemniscus_r	117	-0.31	0.19	0.116	0.969	1.000
posterior_corona_radiata_l	117	-0.08	0.11	0.441	0.969	1.000
medial_lemniscus_l	116	0	0.21	0.982	0.982	1.000
superior_fronto_minus_occipital_fasciculus_could_be_a_part_of_anterior_internal_capsule_l	117	-0.08	0.12	0.531	0.969	1.000
posterior_limb_of_internal_capsule_l	117	-0.21	0.14	0.14	0.969	1.000
anterior_limb_of_internal_capsule_r	117	0.18	0.19	0.356	0.969	1.000
fornix_column_and_body_of_fornix	115	0.2	0.25	0.415	0.969	1.000
superior_longitudinal_fasciculus_r	117	0.14	0.11	0.176	0.969	1.000
retrolenticular_part_of_internal_capsule_r	116	-0.07	0.17	0.673	0.982	1.000
corticospinal_tract_l	115	-0.25	0.21	0.235	0.969	1.000
anterior_corona_radiata_l	116	0.1	0.11	0.374	0.969	1.000
inferior_cerebellar_peduncle_r	116	0.26	0.24	0.293	0.969	1.000
superior_fronto_minus_occipital_fasciculus_could_be_a_part_of_anterior_internal_capsule_r	117	0.18	0.17	0.298	0.969	1.000
posterior_corona_radiata_r	117	-0.13	0.12	0.311	0.969	1.000
external_capsule_r	117	0.14	0.14	0.32	0.969	1.000
body_of_corpus_callosum	117	-0.14	0.19	0.454	0.969	1.000
external_capsule_l	117	-0.1	0.11	0.355	0.969	1.000
superior_corona_radiata_l	117	-0.04	0.12	0.731	0.982	1.000
fornix_cres_stria_terminalis_can_not_be_resolved_with_current_resolution_l	117	0.05	0.11	0.627	0.982	1.000
sagittal_stratum_include_inferior_longitidinal_fasciculus_and_inferior_fronto_minus_occipital_fasciculus_r	117	0.03	0.16	0.866	0.982	1.000
uncinate_fasciculus_r	117	0.01	0.18	0.935	0.982	1.000
retrolenticular_part_of_internal_capsule_l	117	-0.01	0.09	0.911	0.982	1.000
posterior_thalamic_radiation_include_optic_radiation_r	117	0.02	0.12	0.896	0.982	1.000
pontine_crossing_tract_a_part_of_mcp	117	0.11	0.14	0.452	0.969	1.000
cingulum_hippocampus_l	117	-0.12	0.17	0.476	0.969	1.000
superior_longitudinal_fasciculus_l	117	-0.01	0.15	0.933	0.982	1.000
uncinate_fasciculus_l	116	-0.02	0.09	0.837	0.982	1.000
genu_of_corpus_callosum	115	0.09	0.13	0.498	0.969	1.000
posterior_limb_of_internal_capsule_r	117	0.01	0.19	0.95	0.982	1.000
sagittal_stratum_include_inferior_longitidinal_fasciculus_and_inferior_fronto_minus_occipital_fasciculus_l	117	-0.07	0.13	0.559	0.969	1.000
cingulum_cingulate_gyrus_l	117	-0.05	0.18	0.79	0.982	1.000
cerebral_peduncle_l	116	-0.1	0.16	0.553	0.969	1.000
anterior_limb_of_internal_capsule_l	117	-0.11	0.2	0.558	0.969	1.000
anterior_corona_radiata_r	116	0.06	0.1	0.565	0.969	1.000
cingulum_cingulate_gyrus_r	117	0.03	0.12	0.817	0.982	1.000

corticospinal_tract_r	116	0.07	0.25	0.77	0.982	1.000
inferior_cerebellar_peduncle_l	117	-0.03	0.17	0.879	0.982	1.000
cerebral_peduncle_r	116	0.12	0.24	0.634	0.982	1.000
fornix_cres_stria_terminalis_can_not_be_resolved_with_current_resolution_r	117	0.02	0.18	0.921	0.982	1.000
cingulum_hippocampus_r	117	0.02	0.21	0.924	0.982	1.000
superior_cerebellar_peduncle_l	117	-0.02	0.24	0.932	0.982	1.000
superior_cerebellar_peduncle_r	117	0.01	0.19	0.979	0.982	1.000

Supplementary table 6. The effect of insomnia interventions on tract FA. Standerdized effect size (β) for each insomnia intervention compared to the no treatment group at week 7 (T1). FA, fractional anisotropy.

tract	n	CRS					CBT-I				
		β	SE	p	p fdr	p corr	β	SE	p	p fdr	p corr
superior_corona_radiata_r	112	-0.1	0.11	0.346	0.664	1.000	-0.22	0.11	0.057	0.730	1.000
medial_lemniscus_r	114	0.55	0.24	0.026	0.664	1.000	0.43	0.25	0.097	0.730	1.000
corticospinal_tract_l	112	0.39	0.23	0.099	0.664	1.000	0.17	0.25	0.492	0.817	1.000
superior_fronto_minus_occipital_fasciculus_could_be_a_part_of_anterior_internal_capsule_r	113	-0.01	0.12	0.917	0.983	1.000	-0.11	0.12	0.362	0.730	1.000
retrolenticular_part_of_internal_capsule_r	113	0.16	0.17	0.337	0.664	1.000	0.37	0.18	0.040	0.730	1.000
posterior_limb_of_internal_capsule_l	115	0.33	0.17	0.053	0.664	1.000	0.15	0.18	0.393	0.730	1.000
body_of_corpus_callosum	115	0.19	0.16	0.243	0.664	1.000	0.32	0.17	0.067	0.730	1.000
cingulum_cingulate_gyrus_l	116	0.36	0.2	0.070	0.664	1.000	0.34	0.2	0.096	0.730	1.000
middle_cerebellar_peduncle	117	0.29	0.22	0.192	0.664	1.000	0.4	0.23	0.087	0.730	1.000
cingulum_hippocampus_l	116	0.39	0.23	0.095	0.664	1.000	0.24	0.25	0.340	0.730	1.000
fornix_cres_stria_terminalis_can_not_be_resolved_with_current_resolution_r	115	0.29	0.2	0.159	0.664	1.000	0.18	0.21	0.411	0.730	1.000
splenium_of_corpus_callosum	114	-0.13	0.08	0.106	0.664	1.000	-0.08	0.08	0.337	0.730	1.000
superior_fronto_minus_occipital_fasciculus_could_be_a_part_of_anterior_internal_capsule_l	115	0.03	0.22	0.881	0.983	1.000	0.37	0.23	0.109	0.730	1.000
inferior_cerebellar_peduncle_r	114	0.35	0.22	0.120	0.664	1.000	0.3	0.24	0.212	0.730	1.000
superior_cerebellar_peduncle_l	115	0.34	0.23	0.139	0.664	1.000	0.21	0.24	0.379	0.730	1.000
superior_longitudinal_fasciculus_r	114	-0.02	0.07	0.759	0.958	1.000	-0.08	0.08	0.339	0.730	1.000
external_capsule_r	114	-0.06	0.14	0.689	0.918	1.000	-0.14	0.15	0.369	0.730	1.000
pontine_crossing_tract_a_part_of_mcp	117	-0.22	0.16	0.178	0.664	1.000	-0.03	0.16	0.870	0.969	1.000
sagittal_stratum_include_inferior_longitidinal_fasciculus_and_inferior_fronto_minus_occipital_fasciculus_r	115	-0.04	0.15	0.797	0.979	1.000	0.03	0.16	0.838	0.958	1.000
genu_of_corpus_callosum	116	-0.06	0.12	0.630	0.918	1.000	0.14	0.12	0.266	0.730	1.000
superior_corona_radiata_l	113	-0.18	0.14	0.214	0.664	1.000	-0.01	0.15	0.932	0.969	1.000
posterior_thalamic_radiation_include_optic_radiation_l	116	0.2	0.16	0.215	0.664	1.000	0.16	0.17	0.346	0.730	1.000
cingulum_hippocampus_r	115	0.19	0.18	0.288	0.664	1.000	0.07	0.19	0.713	0.863	1.000
cingulum_cingulate_gyrus_r	115	0.16	0.13	0.238	0.664	1.000	0.07	0.14	0.623	0.854	1.000
fornix_cres_stria_terminalis_can_not_be_resolved_with_current_resolution_l	113	-0.01	0.11	0.900	0.983	1.000	0.14	0.12	0.241	0.730	1.000
retrolenticular_part_of_internal_capsule_l	116	-0.13	0.13	0.319	0.664	1.000	-0.13	0.13	0.339	0.730	1.000
superior_longitudinal_fasciculus_l	112	0.15	0.13	0.256	0.664	1.000	0.04	0.14	0.806	0.944	1.000
posterior_thalamic_radiation_include_optic_radiation_r	115	-0.11	0.14	0.435	0.803	1.000	0.06	0.15	0.709	0.863	1.000
external_capsule_l	115	0.02	0.15	0.899	0.983	1.000	0.17	0.15	0.264	0.730	1.000
posterior_corona_radiata_r	112	0.11	0.11	0.286	0.664	1.000	0.12	0.11	0.280	0.730	1.000
medial_lemniscus_l	114	0.21	0.2	0.285	0.664	1.000	0.03	0.21	0.904	0.969	1.000
corticospinal_tract_r	116	-0.26	0.24	0.290	0.664	1.000	-0.14	0.26	0.587	0.854	1.000
no_label_skel	114	0.26	0.24	0.292	0.664	1.000	-0.09	0.26	0.719	0.863	1.000
fornix_column_and_body_of_fornix	116	-0.11	0.23	0.648	0.918	1.000	-0.25	0.24	0.307	0.730	1.000
mean_skel	115	0.09	0.09	0.314	0.664	1.000	0.09	0.09	0.324	0.730	1.000
anterior_corona_radiata_r	115	0.03	0.12	0.816	0.979	1.000	0.11	0.13	0.372	0.730	1.000
cerebral_peduncle_l	114	0	0.14	0.997	1.000	1.000	-0.14	0.15	0.376	0.730	1.000
posterior_corona_radiata_l	116	-0.05	0.15	0.712	0.923	1.000	0.08	0.15	0.603	0.854	1.000
uncinate_fasciculus_r	116	0.1	0.23	0.658	0.918	1.000	0.2	0.24	0.401	0.730	1.000
posterior_limb_of_internal_capsule_r	115	-0.02	0.21	0.921	0.983	1.000	0	0.23	0.994	0.994	1.000
superior_cerebellar_peduncle_r	115	0.08	0.19	0.684	0.918	1.000	0.13	0.2	0.504	0.817	1.000

uncinate_fasciculus_l	114	-0.08	0.13	0.528	0.914	1.000	0.08	0.13	0.511	0.817	1.000
sagittal_stratum_include_inferior_longitidinal_fasciculus_and_inferior_fronto_minus_occipital_fasciculus_l	115	0.09	0.14	0.533	0.914	1.000	-0.01	0.15	0.939	0.969	1.000
cerebral_peduncle_r	115	0	0.23	1.000	1.000	1.000	0.14	0.24	0.550	0.852	1.000
anterior_corona_radiata_l	112	-0.08	0.13	0.552	0.914	1.000	-0.01	0.14	0.949	0.969	1.000
inferior_cerebellar_peduncle_l	116	-0.1	0.18	0.572	0.916	1.000	-0.09	0.19	0.622	0.854	1.000
anterior_limb_of_internal_capsule_r	115	0.1	0.2	0.626	0.918	1.000	0.08	0.21	0.688	0.863	1.000
anterior_limb_of_internal_capsule_l	114	0.01	0.22	0.967	1.000	1.000	0.09	0.24	0.703	0.863	1.000

Supplementary table 7. The effect of insomnia interventions on tract MD. Standerdized effect size (β) for each insomnia intervention compared to the no treatment group at week 7 (T1). MD, mead diffusivity.

		CBT-I+CRS				
**tract**	**n**	β	**SE**	**p**	**p fdr**	**p corr**
superior_corona_radiata_r	112	-0.34	0.11	0.003	0.128	0.108
medial_lemniscus_r	114	0.58	0.25	0.021	0.271	0.832
corticospinal_tract_l	112	0.55	0.24	0.023	0.271	0.910
superior_fronto_minus_occipital_fasciculus_could_be_a_part_of_anterior_internal_capsule_r	113	-0.27	0.12	0.023	0.271	0.911
retrolenticular_part_of_internal_capsule_r	113	0.25	0.17	0.162	0.708	1.000
posterior_limb_of_internal_capsule_l	115	0.29	0.17	0.095	0.708	1.000
body_of_corpus_callosum	115	0.21	0.17	0.218	0.730	1.000
cingulum_cingulate_gyrus_l	116	0.17	0.2	0.388	0.762	1.000
middle_cerebellar_peduncle	117	0.34	0.23	0.138	0.708	1.000
cingulum_hippocampus_l	116	0.15	0.23	0.524	0.838	1.000
fornix_cres_stria_terminalis_can_not_be_resolved_with_current_resolution_r	115	0.35	0.21	0.096	0.708	1.000
splenium_of_corpus_callosum	114	-0.12	0.08	0.131	0.708	1.000
superior_fronto_minus_occipital_fasciculus_could_be_a_part_of_anterior_internal_capsule_l	115	0.2	0.22	0.367	0.762	1.000
inferior_cerebellar_peduncle_r	114	0.22	0.23	0.337	0.762	1.000
superior_cerebellar_peduncle_l	115	0.16	0.23	0.498	0.838	1.000
superior_longitudinal_fasciculus_r	114	-0.11	0.07	0.139	0.708	1.000
external_capsule_r	114	-0.21	0.15	0.161	0.708	1.000
pontine_crossing_tract_a_part_of_mcp	117	-0.11	0.16	0.519	0.838	1.000
sagittal_stratum_include_inferior_longitidinal_fasciculus_and_inferior_fronto_minus_occipital_fasciculus_r	115	-0.21	0.16	0.180	0.718	1.000
genu_of_corpus_callosum	116	-0.16	0.12	0.197	0.728	1.000
superior_corona_radiata_l	113	-0.11	0.14	0.430	0.776	1.000
posterior_thalamic_radiation_include_optic_radiation_l	116	0.15	0.17	0.370	0.762	1.000
cingulum_hippocampus_r	115	-0.22	0.18	0.233	0.730	1.000
cingulum_cingulate_gyrus_r	115	0.02	0.14	0.879	0.995	1.000
fornix_cres_stria_terminalis_can_not_be_resolved_with_current_resolution_l	113	-0.01	0.12	0.918	0.995	1.000
retrolenticular_part_of_internal_capsule_l	116	-0.15	0.13	0.243	0.730	1.000
superior_longitudinal_fasciculus_l	112	0.02	0.14	0.870	0.995	1.000
posterior_thalamic_radiation_include_optic_radiation_r	115	-0.16	0.15	0.262	0.738	1.000
external_capsule_l	115	0.02	0.15	0.893	0.995	1.000

posterior_corona_radiata_r	112	0.1	0.11	0.369	0.762	1.000
medial_lemniscus_l	114	0.2	0.2	0.337	0.762	1.000
corticospinal_tract_r	116	0.02	0.25	0.951	0.995	1.000
no_label_skel	114	0.26	0.25	0.292	0.762	1.000
fornix_column_and_body_of_fornix	116	0.05	0.23	0.846	0.995	1.000
mean_skel	115	0.02	0.09	0.793	0.995	1.000
anterior_corona_radiata_r	115	-0.05	0.12	0.673	0.995	1.000
cerebral_peduncle_l	114	-0.01	0.15	0.940	0.995	1.000
posterior_corona_radiata_l	116	-0.13	0.15	0.397	0.762	1.000
uncinate_fasciculus_r	116	0.01	0.24	0.977	0.995	1.000
posterior_limb_of_internal_capsule_r	115	-0.17	0.21	0.437	0.776	1.000
superior_cerebellar_peduncle_r	115	-0.01	0.19	0.959	0.995	1.000
uncinate_fasciculus_l	114	-0.01	0.13	0.967	0.995	1.000
sagittal_stratum_include_inferior_longitidinal_fasciculus_and_inferior_fronto_minus_occipital_fasciculus_l	115	0	0.14	0.995	0.995	1.000
cerebral_peduncle_r	115	-0.14	0.23	0.556	0.860	1.000
anterior_corona_radiata_l	112	-0.04	0.13	0.737	0.995	1.000
inferior_cerebellar_peduncle_l	116	-0.02	0.19	0.906	0.995	1.000
anterior_limb_of_internal_capsule_r	115	-0.07	0.2	0.721	0.995	1.000
anterior_limb_of_internal_capsule_l	114	0	0.22	0.988	0.995	1.000

Supplementary table 7. The effect of insomnia interventions on tract MD. Standerdized effect size (β) for each insomnia intervention compared to the no treatment group at week 7 (T1). MD, mead diffusivity.
